# Supplementary material for: A new tool for converting food frequency questionnaire data into nutrient and food group values: FETA research methods and availability
Source: BMJ Open. 2014 Mar 27;4(3):e004503. doi: 10.1136/bmjopen-2013-004503 (PMC3975761; doi:10.1136/bmjopen-2013-004503)
Supplement: Author's manuscript [file bmjopen-2013-004503.draft_revisions.pdf]

A new tool for converting food frequency questionnaire data into nutrient and food group values: FETA research methods and availability

Journal:	*BMJ Open*
Manuscript ID:	bmjopen-2013-004503
Article Type:	Research
Date Submitted by the Author:	19-Nov-2013
Complete List of Authors:	Mulligan, Angela; University of Cambridge, Public Health & Primary Care<br>Luben, Robert; University of Cambridge, Public Health & Primary Care<br>Bhaniani, Amit; University of Cambridge, Public Health & Primary Care<br>Parry-Smith, David; University of Cambridge, Public Health & Primary Care<br>O'Connor, Laura; University of Cambridge, Institute of Metabolic Science<br>Khawaja, Anthony; University of Cambridge, Public Health & Primary Care<br>Forouhi, Nita; University of Cambridge, Institute of Metabolic Science<br>Khaw, KayTee; University of Cambridge, Clinical Medicine
**Primary Subject Heading**:	Research methods
Secondary Subject Heading:	Health informatics, Public health, Nutrition and metabolism, Epidemiology
Keywords:	PUBLIC HEALTH, EPIDEMIOLOGY, Nutrient intakes, food frequency questionnaire

SCHOLARONE™
Manuscripts

A new tool for converting food frequency questionnaire data into nutrient and food group values: FETA research methods and availability

Angela A Mulligan[a1 c1], Robert N Luben[a1], Amit Bhaniani[a1], David J Parry-Smith[a1], Laura O'Connor[a2], Anthony P Khawaja[a1], Nita G Forouhi*[a2], Kay-Tee Khaw*[a1, a3]

* indicates equal contribution as authors

[a1] European Prospective Investigation into Cancer and Nutrition, Department of Public Health and Primary Care, University of Cambridge, Strangeways Research Laboratory, Worts Causeway, Cambridge, UK

[a2] MRC Epidemiology Unit, Institute of Metabolic Science, Addenbrooke's Hospital, University of Cambridge, Cambridge, UK

[a3] EPIC, Department of Gerontology, Addenbrooke's Hospital, School of Clinical Medicine, University of Cambridge, Cambridge, UK

Source of support: MRC Population Health Sciences Research Network (PHSRN), Cancer Research UK (C864/A8257) and the Medical Research Council (G0401527, G1000143)

Running title: FETA: new processing tool for FFQs

Key words: food frequency questionnaire, nutritional output, processing tool, EPIC-Norfolk

Correspondence:

[c1] Corresponding author: Ms A. Mulligan, telephone +44 1223 748683, fax +44 1223 748676, email angela.mulligan@phpc.cam.ac.uk

Abbreviations: FFQ, food frequency questionnaire; EPIC, European Prospective Investigation into Cancer and Nutrition; FETA, FFQ EPIC Tool for Analysis; CAFÉ, Compositional Analyses from Frequency Estimates

Word count: 3 254

For peer review only

ABSTRACT

Objectives

To describe the research methods for the development of a new tool which processes data from the European Prospective Investigation into Cancer and Nutrition Norfolk Food Frequency Questionnaire (EPIC-Norfolk FFQ). A further aim was to compare nutrient and food group values derived from the current tool (FETA; FFQ EPIC Tool for Analysis) with the previously validated but less accessible tool, CAFÉ (Compositional Analyses from Frequency Estimates). The effect of text matching on intake data was also investigated

Design

Cross-sectional analysis of a prospective cohort study – EPIC-Norfolk.

Setting

East England population (city of Norwich and its surrounding small towns and rural areas).

Participants

Complete FFQ data from 11 250 men and 13 602 women with a mean age of 59 years (range 40 – 79 years).

Outcome measures

Nutrient and food group intakes derived from FETA and CAFÉ analyses of EPIC-Norfolk FFQ data.

Results

Nutrient outputs from FETA and CAFÉ were similar; mean (SD) energy intake from FETA was 9222 kJ (2633) in men, 8113 kJ (2296) in women, compared to CAFÉ intakes of 9175 kJ (2630) in men, 8091 kJ (2298) in women. The majority of differences resulted in one or less quintile change (98.7%). Only mean daily fruit and vegetable food group intakes were higher in women than in men (278 v 212 g and 284 v 255 g respectively). Quintile changes were evident for all nutrients, with the exception of alcohol, when text matching was not executed; however, only the cereals food group was affected.

For peer review only

Conclusions

FETA produces similar nutrient and food group values to the previously validated CAFÉ but has the advantages of being open source, cross-platform and complete with a data-entry form directly compatible with the software. The tool will facilitate research using the EPIC-Norfolk FFQ, and can be customised for different study populations.

Strengths and limitations of this study

- FETA has been tested using a large study sample of food intake data.
- No independent reference method used in the comparisons of Feta and CAFÉ nutrient intake data although the CAFÉ system has been previously validated.

INTRODUCTION

Food Frequency Questionnaires (FFQs) are commonly used in epidemiological studies to assess the dietary intake of large populations. Their popularity derives from ease of administration, ability to assess dietary intake over a defined period of time, and low costs (1). The European Prospective Investigation into Cancer and Nutrition (EPIC)-Norfolk FFQ is semi-quantitative and designed to record the average intake of foods during the previous year. The principles involved in data collection and processing of the EPIC-Norfolk FFQ and the development of the structure and content of the CAFÉ program for calculating nutrient intakes have been published previously (2). The EPIC-Norfolk FFQ has been extensively validated and has been widely used (3);(4);(5). However, the programs used to process these FFQs, including CAFÉ, have not been easily accessible to end-users.

Our objectives were to develop a new, open source, cross-platform processing tool (FETA - FFQ EPIC Tool for Analysis) based on and building upon the earlier system, CAFÉ (2). The aim of this report was to describe the research methods of the development of FETA, and to compare nutrient output from the FETA and CAFÉ programs. Food group intake data from FETA has also been described as has the effect of free text matching on nutrient and food group intake data.

For peer review only

METHODS

EPIC-FFQ design

The questionnaire consists of two parts. Part 1 consists of a food list of 130 lines; each line has a portion size attached to it: medium serving, standard unit or household measure. Study participants were requested to select an appropriate frequency of consumption for each line, from the nine frequency categories. As an example, Figure 1 illustrates the sections relating to bread, savoury biscuits and breakfast cereals. A pdf copy of the EPIC-Norfolk FFQ may be downloaded from http://www.srl.cam.ac.uk/epic/epicffq/websitedocumentation.html; information on how to complete and code the FFQ is also available here. The questionnaire lines are either individual foods, combinations of individual foods or food types. The FFQ food list is based on items from an FFQ widely used within the USA (6);(7), but modified to reflect differences in American versus UK brand names and some further food items were added.

Part 2 contains further questions, a number of which ask for more detailed information that link back to food lines in part one, as illustrated in Figure 2. Detailed information was requested for breakfast cereals and fats as these are nutritionally important foods in the UK diet.

Data collection

The EPIC-Norfolk FFQ was posted to 25 639 participants in the EPIC-Norfolk cohort study (8). The participants were aged 40-79 years and the questionnaire was completed between 1993 and 1997. The study was approved by the Norfolk Local Research Ethics Committee, adhered to the Declaration of Helsinki and all participants gave written informed consent. The FFQ was returned at a health examination, where it was checked and completed, if required, by trained nursing staff. In total, 25 351 (99%) participants returned the completed questionnaire.

Comparison of FETA and CAFÉ programs

FETA uses a csv (comma-separated values) input file. Part 1 is coded as numeric values and Part 2 is coded as numeric values and food codes, using the flow-charts and look-up lists provided

For peer review only

(http://www.srl.cam.ac.uk/epic/epicffq/). We have also created a Microsoft Access form-based entry tool to facilitate FFQ data entry, based on the EPIC-Norfolk FFQ. The tool exports data in a format directly compatible with FETA. The FETA software was written in C and C++ languages, enabling faster processing times than SAS and the C/C++ software can also be used from the command line. The step-based graphical wizard for running FETA was written in Perl. Whereas in the CAFÉ program, an Oracle (Oracle Corporation, Redwood Shores, CA, USA) -based entry system was created to enter Part 1 frequency data as numeric codes and Part 2 data as numeric codes and free text. CAFÉ was written using SAS (SAS Software, Version 8 of the SAS System for Unix, SAS Institute Inc., Cary, NC, USA) and links to tables in an Oracle relational database.

Part 1- data entry

Data were manually entered into a spreadsheet as numeric codes, using '1' for 'never or less than once a month', to '9' for '6+ times per day'. A code of '-9' was used to mark data where a frequency was not recorded. Where two frequencies were provided for a line, these were both coded, separated by a semi-colon, e.g. '2;3', and FETA processed the first value. In the CAFÉ program, two entries per line were treated as missing data.

Part 2 – assigning of food codes to ticked boxes and free text

Part 2 contains hand-written text for milk, breakfast cereals and cooking fats (see Figure 2, questions 3, 5, 6 and 7 respectively), which needs to be matched to the most appropriate food code in order to obtain nutrient data; this process is known as free text matching. The data in part 2 were coded using reference lists of food codes for varieties of milk, breakfast cereal and cooking fat. Where there is no clear match, it is suggested that a researcher consults the ingredients and nutrient information of the commercial item and compares this information with the nutrient profile of similar items from the reference lists. These reference lists and figures relating to food codes that may be assigned to appropriate ticked boxes may be found at http://www.srl.cam.ac.uk/epic/epicffq/websitedocumentation.html

For peer review only

Differences between FETA versus CAFÉ processing may also be found at http://www.srl.cam.ac.uk/epic/epicffq/websitedocumentation.html; these differences relate to breakfast cereals, frying and baking fats, the outcome of selecting the 'None' or 'No' box, and default milk, cereal, and fat codes.

Databases

Each line in Part 1 of the FFQ is mapped to up to six food codes. Decisions regarding which food codes to use were based on data from UK government surveys and other UK population data (7); (7,9,10). These decisions were based on data for individuals aged 40-74 years (7). Data for portion weights were sourced from UK population data and weighed records in 40-74 year old study participants (7,11).

The EPIC-Norfolk FFQ uses 290 foods from the UK food composition database, McCance and Widdowson's "The Composition of Foods" (5th edition) and its associated supplements (12–21). A number of new food items were added to the EPIC-Norfolk FFQ food list, which are used in both in FETA and CAFÉ programs. These include low calorie/diet fizzy drinks and crunchy oat cereal, as well as modified home-baked and fried foods (without their fat), to enable an individual's fat type, as recorded in Part 2 of the FFQ, to be incorporated. However, the nutrient data of six of the nine new foods used in the CAFÉ program were modified in FETA. These foods include crunchy oat cereal, milk non-specific, low calorie/diet fizzy drinks, solid vegetable oil, Crisp 'n Dry (solid fat), and oil and fat non-specific. Modifications to the nutrient data were made to ensure a more accurate nutrient profile and/or to better reflect the foods consumed, in the case of non-specific items, such as milk and oil/fat.

Identification of outliers

Outliers were defined, as detailed previously (2). In brief, the ratio of energy intake (EI) to basal metabolic rate (BMR) was calculated, where BMR was calculated using sex-specific Schofield equations, which included age and body weight (22). Individuals in the top and bottom 0.5% of EI:

For peer review only

BMR ratio were identified and excluded, as were individuals with FFQs containing 10 or more missing lines of data in Part 1of the FFQ.

Nutrient and food group outputs

FETA produces four nutrient output formats and a sample of each of these can be viewed at http://www.srl.cam.ac.uk/epic/epicffq/websitedocumentation.html

Output 1 contains average daily nutrient and food group intakes for an individual from all FFQ foods consumed, in wide format, suitable for import into a spreadsheet or statistical package. Intake data for 46 nutrients are provided as well as data for 14 basic food groups, however only a selection of these nutrients is shown in this report. Output 2 contains the same nutrient intake data as output 1, but in long format, which is mostly suitable for programmers. Output 3 contains average daily nutrient and food group intakes (and amount of food consumed) for an individual for each FFQ line; this output file will be very large and is mostly suitable for programmers. The most detailed output (output 4) contains average daily nutrient and food group intakes, in addition to the amount of food consumed for an individual, for each food code, for each FFQ line (meal_id). An online description of each meal_id and nutrient code, including units of measurement, can be found in the data entry template. This output will also be very large and is mostly suitable for programmers.

A log file is created along with each output file, which records the processing of the data and provides useful error information (see Appendix 1 for log file of output 1). In these files, both notes (general process information) and error messages are recorded, with a date and time stamp. The log files make it possible to calculate the number of missing frequencies based on Part 1 (main grid) of the FFQ in order to exclude individuals with 10 or more missing ticks. The log files also record situations where a food code does not have any nutrient data attached to it.

Statistical analyses

The data were analysed using STATA 10 (STATA Corp., Texas, USA). Intake data were described using mean, standard deviation (SD), median, minimum and maximum for both FETA and CAFÉ program outputs, stratified by sex. The nutrients selected for comparison are those described in the

For peer review only

original CAFÉ paper. Where data on quintile changes are shown, cut-off points were calculated using CAFÉ nutrient data in order to compare quintile shift between FETA and CAFÉ output data.

RESULTS

There were FFQ data available from 25 351 participants with a mean age of 59 years. Data from 11 250 men and 13 602 women are presented here, as individuals in the top and bottom 0.5% of EI: BMR ratio have been excluded, as have individuals with FFQs containing 10 or more missing lines of data in Part 1of the FFQ.

Nutrient intake data from FETA and CAFÉ programs

Table 1 shows the average daily intake data for a number of selected nutrients for 11 250 men. The data were similar for most nutrients across the two programs. The nutrients which had the highest percentage of quintile change (≥10%) were monounsaturated fat, saturated fat, iron, vitamin D & vitamin E. However, only 1.3% of the men changed more than one quintile, for two of these five nutrients. The nutrients which had the lowest percentage of quintile changes were alcohol, calcium and carotene, with less than 3% change (Table 1).

Table 2 shows average daily intake data for the selected nutrients for 13 602 women, from FETA and CAFÉ programs. There were similar quintile changes observed in women to those found in men for the selected nutrients; four of the nineteen nutrients had a quintile change of greater than 10%: polyunsaturated fat, saturated fat, iron and Vitamin E. However, the number of women who shifted more than one quintile was generally lower than the number observed in men. The nutrients which had the greatest percentage of women who changed more than one quintile were vitamins D and E, with 0.7 and 0.9% respectively.

Detailed (output 4) nutrient intake data at the individual level obtained from the two programs were compared for approximately half of the participants (n=12 500; data not shown). All differences (> 0.1%) found were investigated and explanations for these differences are considered in the discussion.

For peer review only

Table 1 Average daily nutrient intakes for men (N=11 250) participating in the EPIC-Norfolk study, from the FETA and CAFÉ programs, after the exclusion of outliers, with numbers and percentages of men who moved quintile

	FETA program					CAFÉ program								
Nutrient	Median	Mean	SD	Minimum	Maximum	Median	Mean	SD	Minimum	Maximum	Quintile change		Quintile change > 1	
											N	%	N	%
Energy (kcals)	2126	2190	627	748	5085	2115	2179	626	748	5101	892	7.9	0	0.0
Energy (kJs)	8947	9222	2633	3124	21394	8900	9175	2630	3124	21440	891	7.9	0	0.0
Protein (g)	83.4	85.2	22.0	23.3	319.8	83.2	84.9	22.0	23.3	318.4	464	4.1	0	0.0
Alcohol (g)	6.7	12.3	16.1	0.0	134.2	6.7	12.3	16.1	0.0	134.2	0	0.0	0	0.0
Carbohydrate (g)	261	271	87	48	737	259	269	87	48	729	726	6.5	0	0.0
Starch (g)	123	128	45	10	504	122	127	45	10	501	813	7.2	1	0.0
Englyst fibre (g)	17.5	18.2	6.4	1.3	89.9	17.3	18.0	6.4	1.3	89.9	743	6.6	1	0.0
Fat (g)	78.9	83.2	31.3	13.4	260.6	78.7	83.0	31.3	13.4	260.6	1049	9.3	8	0.1
Monounsaturated fat (g)	27.0	28.8	11.6	4.8	101.2	26.8	28.5	11.5	4.8	105.1	1264	11.2	21	0.2
Polyunsaturated fat (g)	13.5	15.0	6.9	1.6	66.6	13.7	15.3	7.1	1.6	69.5	1074	9.5	24	0.2
Saturated fat (g)	30.1	32.3	13.6	3.0	110.6	29.8	31.9	13.5	3.0	106.7	1288	11.5	20	0.2
Calcium (mg)	1021	1039	301	189	2848	1018	1037	300	189	2849	296	2.6	1	0.0
Iron (mg)	12.1	12.4	3.6	2.6	38.7	11.9	12.3	3.5	2.5	38.5	1149	10.2	7	0.1
Potassium (mg)	3814	3881	911	1305	11718	3802	3869	909	1284	11718	411	3.7	0	0.0

For peer review only

Carotene (mcg)	3188	3321	1573	147	25720	3178	3309	1571	147	25720	156	1.4	0	0.0
Folate (mcg)	320	331	97	77	1547	316	327	96	77	1547	836	7.4	3	0.0
Vitamin C (mg)	103	111	52	10	669	105	113	52	10	669	411	3.7	14	0.1
Vitamin D (mcg)	3.16	3.65	2.08	0.03	27.08	3.13	3.62	2.06	0.03	27.12	1161	10.3	145	1.3
Vitamin E (mg)	13.2	14.9	7.2	2.1	62.3	12.9	14.4	6.8	2.1	62.0	1545	13.7	146	1.3

For peer review only

Table 2 Average daily nutrient intakes for women (N=13 602) participating in the EPIC-Norfolk study, from the FETA and CAFÉ programs, after the exclusion of outliers, with numbers and percentages of women who moved quintile

Nutrient	FETA program Median	Mean	SD	Minimum	Maximum	CAFÉ program Median	Mean	SD	Minimum	Maximum	Quintile change N	%	Quintile change > 1 N	%
Energy (kcals)	1859	1925	546	538	4733	1853	1920	547	518	4643	1030	7.6	0	0.0
Energy (kJs)	7833	8113	2296	2261	19910	7811	8091	2298	2179	19537	1018	7.5	0	0.0
Protein (g)	79.8	81.5	21.1	23.0	246.0	79.6	81.3	21.0	22.7	246.1	495	3.6	1	0.0
Alcohol (g)	2.0	5.6	8.4	0.0	99.5	2.0	5.6	8.4	0.0	99.5	0	0.0	0	0.0
Carbohydrate (g)	237	247	77	59	766	235	245	77	58	766	974	7.2	1	0.0
Starch (g)	107	112	39	13	405	106	111	39	13	406	1142	8.4	1	0.0
Englyst fibre (g)	18.2	19.0	6.8	2.3	118.5	18.0	18.8	6.7	2.4	118.6	850	6.2	1	0.0
Fat (g)	67.0	70.8	27.1	11.7	221.0	67.2	71.2	27.3	11.6	217.2	1194	8.8	4	0.0
Monounsaturated fat (g)	22.5	24.1	9.9	3.8	100.3	22.5	24.1	9.9	3.5	100.6	1338	9.8	7	0.1
Polyunsaturated fat (g)	12.2	13.5	6.2	2.0	53.6	12.5	13.8	6.3	2.0	53.6	1434	10.5	23	0.2
Saturated fat (g)	25.0	27.0	11.7	3.6	102.3	25.0	26.9	11.7	3.7	99.3	1443	10.6	9	0.1
Calcium (mg)	971	992	290	128	3159	969	990	290	127	3159	390	2.9	4	0.0
Iron (mg)	11.5	11.8	3.6	1.7	66.1	11.3	11.7	3.5	1.8	65.7	1496	11.0	12	0.1
Potassium (mg)	3781	3861	942	1150	16568	3769	3848	939	1147	16587	486	3.6	1	0.0
Carotene (mcg)	3477	3719	1917	67	61971	3469	3712	1917	64	61983	122	0.9	0	0.0

For peer review only

Folate (mcg)	322	332	103	65	2039	317	328	101	65	2024	1025	7.5	5	0.0
Vitamin C (mg)	123	133	64	4	1006	125	135	64	4	1006	746	5.5	35	0.3
Vitamin D (mcg)	3.01	3.46	1.90	0.00	17.83	3.02	3.45	1.90	0.00	17.75	1119	8.2	90	0.7
Vitamin E (mg)	12.4	13.8	6.2	1.5	52.4	12.2	13.5	6.0	1.6	49.8	1863	13.7	123	0.9

For peer review only

Food group intake data from FETA

Average daily intakes for both men and women of the fourteen food groups readily available from FETA are shown in Table 3. Mean daily intakes of six of the food groups were higher in men than in women: alcohol, cereals, fats, meat, potatoes and sugars. However, women had higher intakes of fruit (278g v 212g) and vegetables (284g v 25+5g). Mean daily intakes of eggs, fish, milk, non-alcoholic beverages, nuts and seeds, and soups and sauces were similar in both men and women.

The effect of text matching in FETA

Tables 4 and 5 illustrate the variation in nutrient and food group intake data obtained in a random subset of 1 159 men and 1 340 women, respectively, depending on whether text matching of milks, breakfast cereals and baking and frying fats was applied. In general, mean nutrient intakes were higher when text matching was carried out. In men, (Table 4), quintile changes (>15%) were most evident in the following nutrients: Englyst fibre, polyunsaturated fat, folate, vitamin D and vitamin E. The food group "cereals and cereal products" was the only one of the fourteen groups where there was a difference, with 31 men moving 1 quintile.

In women, (Table 5), quintile changes (>15%) were also most evident in the same five nutrients. However, almost 21% of women also changed quintile for iron. Once again, the "cereals and cereal products" food group was the only food group where there was any difference, with 40 women moving 1 quintile.

For peer review only

Table 3 Average daily food group intakes for men (N=11 250) and women (N=13 602) participating in the EPIC-Norfolk study, from the FETA program

	**Men**					**Women**				
Food group	Median	Mean	SD	Minimum	Maximum	Median	Mean	SD	Minimum	Maximum
Alcoholic beverages (g)	101	204	315	0	2483	23	64	109	0	1728
Cereals & cereal products (g)	242	260	127	0	1456	215	231	110	0	1172
Eggs & egg dishes (g)	18	17	15	0	225	14	16	14	0	236
Fats & oils (g)	31	36	22	0	207	27	30	20	0	218
Fish & fish products (g)	32	37	26	0	362	32	38	26	0	309
Fruit (g)	179	212	164	0	2654	238	278	201	0	3742
Meat & meat products (g)	99	106	54	0	856	91	94	48	0	606
Milk & milk products (g)	407	420	182	0	1303	386	410	175	0	1560
Non-alcoholic beverages (g)	1157	1177	396	0	3707	1150	1165	403	0	4501
Nuts & seeds (g)	0	3	9	0	228	0	3	9	0	188
Potatoes (g)	125	122	69	0	1007	116	112	64	0	1506
Soups & sauces (g)	43	58	54	0	1004	43	57	53	0	1376
Sugars (g)	53	64	50	0	572	37	48	42	0	541
Vegetables (g)	236	255	123	0	2398	262	284	143	0	3539

For peer review only

Table 4 Comparison of average daily nutrient and food group intakes for men (N=1 159) participating in the EPIC-Norfolk study, from the FETA program, with and without the application of text matching

	**FETA program, with text matching**					**FETA program, without text matching**								
Nutrient/Food group	Median	Mean	SD	Minimum	Maximum	Median	Mean	SD	Minimum	Maximum	Quintile change		Quintile change > 1	
											N	%	N	%
Energy (kcals)	2095	2176	678	658	7766	2091	2170	678	658	7787	28	2.4	0	0.0
Energy (kJs)	8822	9161	2848	2780	32555	8804	9138	2850	2780	32647	26	2.2	0	0.0
Protein (g)	82.8	85.0	22.8	22.1	272.3	82.5	84.7	22.8	22.1	272.3	34	2.9	0	0.0
Alcohol (g)	7.2	12.3	16.1	0.0	112.9	7.2	12.3	16.1	0.0	112.9	0	0.0	0	0.0
Carbohydrate (g)	261	270	93	63	1006	259	269	93	63	1003	48	4.1	0	0.0
Starch (g)	120	127	49	7	643	121	126	48	7	636	65	5.6	0	0.0
Englyst fibre (g)	17.5	18.3	6.6	3.6	71.8	17.3	17.9	6.3	3.6	64.5	198	17.1	10	0.9
Fat (g)	77.8	82.1	33.1	12.8	387.8	77.3	82.1	33.1	12.8	389.3	32	2.8	0	0.0
Monounsaturated fat (g)	26.5	28.2	12.2	3.5	131.1	26.7	28.7	12.5	3.7	138.7	88	7.6	0	0.0
Polyunsaturated fat (g)	13.5	14.9	7.3	3.0	67.0	12.7	14.1	6.8	3.0	60.7	179	15.4	17	1.5
Saturated fat (g)	30.1	31.8	14.1	3.3	160.0	30.3	32.2	14.3	3.3	160.3	72	6.2	1	0.1
Calcium (mg)	1015	1044	312	242	2848	1012	1044	313	242	2861	42	3.6	0	0.0
Iron (mg)	11.9	12.5	3.8	2.6	37.9	11.7	12.0	3.5	2.6	38.1	173	14.9	16	1.4
Potassium (mg)	3824	3889	957	1353	12675	3812	3873	951	1353	12551	52	4.5	0	0.0

For peer review only

Carotene (mcg)	3150	3348	1671	507	18295	3162	3353	1672	507	18338	6	0.5	0	0.0
Folate (mcg)	325	333	103	94	1222	316	326	101	94	1262	226	19.5	2	0.2
Vitamin C (mg)	105	113	55	17	619	104	112	55	17	619	22	1.9	0	0.0
Vitamin D (mcg)	3.08	3.64	2.17	0.03	16.40	3.06	3.64	2.19	0.03	20.52	227	19.6	8	0.7
Vitamin E (mg)	13.3	15.0	7.6	2.7	74.7	13.0	14.5	7.1	2.7	71.2	238	20.5	30	2.6
Alcoholic beverages (g)	104	201	301	0	1866	104	201	301	0	1866	0	0.0	0	0.0
Cereals & cereal products (g)	240	257	131	0	1378	238	255	130	0	1378	31	2.7	0	0.0
Eggs & egg dishes (g)	18	17	17	0	225	18	17	17	0	225	0	0.0	0	0.0
Fats & oils (g)	31	36	25	0	313	31	36	25	0	313	0	0.0	0	0.0
Fish & fish products (g)	32	37	25	0	153	32	37	25	0	153	0	0.0	0	0.0
Fruit (g)	184	216	158	0	1037	184	216	158	0	1037	0	0.0	0	0.0
Meat & meat products (g)	98	104	52	0	690	98	104	52	0	690	0	0.0	0	0.0
Milk & milk products (g)	414	428	187	0	1302	414	428	187	0	1302	0	0.0	0	0.0
Non-alcoholic beverages (g)	1159	1191	397	22	3677	1159	1191	397	22	3677	0	0.0	0	0.0
Nuts & seeds (g)	0	3	8	0	135	0	3	8	0	135	0	0.0	0	0.0
Potatoes (g)	125	121	78	0	1518	125	121	78	0	1518	0	0.0	0	0.0
Soups & sauces (g)	43	56	51	0	556	43	56	51	0	556	0	0.0	0	0.0
Sugars (g)	51	63	50	0	358	51	63	50	0	358	0	0.0	0	0.0
Vegetables (g)	238	256	128	15	1047	238	256	128	15	1047	0	0.0	0	0.0

For peer review only

Table 5 Comparison of average daily nutrient and food group intakes for women (N=1 340) participating in the EPIC-Norfolk study, from the FETA program, with and without the application of text matching

	**FETA program, with text matching**					**FETA program, without text matching**								
Nutrient/Food group	Median	Mean	SD	Minimum	Maximum	Median	Mean	SD	Minimum	Maximum	Quintile change		Quintile change > 1	
											N	%	N	%
Energy (kcals)	1886	1946	607	608	8103	1880	1941	605	608	8134	50	3.7	0	0.0
Energy (kJs)	7938	8202	2554	2552	34410	7909	8177	2547	2552	34541	47	3.5	0	0.0
Protein (g)	80.3	82.5	22.2	26.8	277.0	79.9	82.1	22.1	26.8	276.6	43	3.2	0	0.0
Alcohol (g)	2.0	5.4	8.1	0.0	65.3	2.0	5.4	8.1	0.0	65.3	0	0.0	0	0.0
Carbohydrate (g)	238	250	90	67	1596	237	249	90	67	1603	58	4.3	0	0.0
Starch (g)	109	114	52	25	1288	108	114	52	25	1301	99	7.4	0	0.0
Englyst fibre (g)	18.6	19.3	7.4	4.1	103.7	17.8	18.7	7.1	3.3	97.2	247	18.4	13	1.0
Fat (g)	67.6	71.4	28.5	17.2	259.4	67.5	71.3	28.4	17.2	259.7	45	3.4	0	0.0
Monounsaturated fat (g)	22.7	24.4	10.6	4.8	104.2	23.1	24.6	10.6	4.8	103.8	133	9.9	0	0.0
Polyunsaturated fat (g)	12.2	13.6	6.2	2.6	42.5	11.5	12.9	5.9	2.5	39.4	224	16.7	11	0.8
Saturated fat (g)	25.2	27.2	12.4	5.1	109.6	25.5	27.5	12.4	5.1	109.6	74	5.5	2	0.1
Calcium (mg)	978	995	298	242	2528	976	992	297	242	2534	46	3.4	1	0.1
Iron (mg)	11.7	11.9	3.9	3.1	67.8	11.1	11.4	3.5	3.1	55.3	280	20.9	44	3.3
Potassium (mg)	3788	3874	994	1284	12702	3744	3848	987	1280	12526	68	5.1	0	0.0

For peer review only

Carotene (mcg)	3489	3731	1705	178	13796	3500	3736	1707	175	13796	11	0.8	0	0.0
Folate (mcg)	326	337	107	102	1311	318	329	105	97	1276	291	21.7	1	0.1
Vitamin C (mg)	124	133	63	4	809	122	132	62	4	809	34	2.5	0	0.0
Vitamin D (mcg)	3.07	3.49	1.89	0.22	12.06	3.02	3.46	1.89	0.29	12.46	248	18.5	9	0.7
Vitamin E (mg)	12.5	13.8	6.3	2.7	52.4	12.1	13.3	5.9	3.3	43.6	270	20.2	21	1.6
Alcoholic beverages (g)	21	61	104	0	1350	21	61	104	0	1350	0	0.0	0	0.0
Cereals & cereal products (g)	214	236	174	9	4948	212	234	174	9	4948	40	3.0	0	0.0
Eggs & egg dishes (g)	14	16	14	0	136	14	16	14	0	136	0	0.0	0	0.0
Fats & oils (g)	27	30	19	0	133	27	30	19	0	133	0	0.0	0	0.0
Fish & fish products (g)	32	39	26	0	187	32	39	26	0	187	0	0.0	0	0.0
Fruit (g)	238	277	199	0	2830	238	277	199	0	2830	0	0.0	0	0.0
Meat & meat products (g)	90	95	49	0	392	90	95	49	0	392	0	0.0	0	0.0
Milk & milk products (g)	381	410	174	0	959	381	410	174	0	959	0	0.0	0	0.0
Non-alcoholic beverages (g)	1148	1153	404	8	3215	1148	1153	404	8	3215	0	0.0	0	0.0
Nuts & seeds (g)	0	3	11	0	180	0	3	11	0	180	0	0.0	0	0.0
Potatoes (g)	116	113	61	0	785	116	113	61	0	785	0	0.0	0	0.0
Soups & sauces (g)	45	57	53	0	900	45	57	53	0	900	0	0.0	0	0.0
Sugars (g)	38	50	46	0	540	38	50	46	0	540	0	0.0	0	0.0
Vegetables (g)	265	288	140	2	1387	265	288	140	2	1387	0	0.0	0	0.0

For peer review only

DISCUSSION

FETA provides a new, freely available, standalone tool that can produce nutrient and food group intake values from data collected using the EPIC-Norfolk FFQ. It makes the EPIC-Norfolk FFQ readily accessible to end-users and enables them to process and analyse nutritional data. The data can either be entered into a spreadsheet, using the instructions provided, or by using the specifically developed Microsoft Access form-based entry tool. The Access entry tool allows easier entry without requiring knowledge of specific food codes. The software for FETA for Windows and Linux can be downloaded from the website, as can the Microsoft Access data entry utility (http://www.srl.cam.ac.uk/epic/epicffq/). Users are encouraged to register with EPIC-Norfolk, as this enables them to request assistance and support. The various types of output (with four levels of information) available should prove beneficial to researchers, especially those requiring more detailed information. There is an on-going need for information on the intake of food groups. While the data from either output 3 or 4 could be used to generate more detailed food group data, we have treated food groups as another type of nutrient – a pseudo-nutrient. The FETA input/look-up files can be easily modified to create new groups, greatly adding to the flexibility of the system for analysing food group consumption, while requiring no spreadsheet or programming skills on the part of the analyst. A helpful feature of FETA is the log file which documents errors relating to FFQ data and/or default food codes assigned.

FETA was designed and based on the extensively validated EPIC-Norfolk FFQ, originally developed in 1988, to assess the nutrient and food group intake of 40-79 year olds, who completed the FFQ between 1993 and 1997. The food list and look-up lists of milks, breakfast cereals and fats reflect this time period and the study population, as do the default milk, cereal, baking fat and frying fat codes assigned. However, the program was created in such a way that it can be customised for different study populations, easily enabled by the separation of the processing algorithm in the FETA program implementation from the data model text files. It is possible to delete/add foods and/or FFQ lines, and modify portion sizes as desired for a study.

For peer review only

Comparisons were carried out for a number of selected nutrients obtained from FETA and the previously validated CAFÉ program. These showed that the nutrient output from both programs were generally similar. All differences (>0.1%) found from the comparison of detailed food/nutrient data at the individual level for 12 500 participants from FETA and the CAFÉ program can be explained by one or more of the following reasons: up to four cereal foods assigned by FETA, as compared to a maximum of two cereal foods assigned by CAFÉ; differences in default baking and frying fat codes assigned; correction for muesli portion size in cereal data; exclusion of porridge from cereal data (free text); default codes assigned for milk, cereals or fats to participants using FETA (where no food codes were assigned by CAFÉ program); rounding error (only where percentage absolute differences were between 0.1 to 1%) and changes made to the nutrient data of six of the nine new foods as well as to the default code for milk. Although nutrient intakes as calculated by FETA and CAFÉ were similar, some relatively small differences existed, but these and the quintile shift of men and women can be explained. In FETA, a number of changes were made to the processing of breakfast cereals, affecting carbohydrate, starch, Englyst fibre, iron and folate estimates. The vitamin C content per 100g of low calorie/diet fizzy drinks was changed from 5 to 0 mg and the vitamin E content of crunchy oat cereal and oil and fat non-specific was increased. Changes made to the processing of fats in Questions 6 and 7 in Part 2 of the FFQ, in addition to changes made to the fatty acid profile of the three new fats, could help explain the small differences observed in monounsaturated, polyunsaturated and saturated fat intakes.

There was quite a large range in intake in the fourteen food groups, with a minimum intake of zero for each of the food groups. It is difficult to compare food group intake data as the groupings of foods often varies. However, the combined mean intake of fruit (excluding juices) and vegetables for men and women was 467g and 562g respectively, achieving the Government's 'Five a day' recommendation(23), using a portion size of 80 g.

For peer review only

Whilst text matching only affected one food group (cereals and cereal products), more than 15% of men and women changed quintile for a number of nutrients: Englyst fibre, polyunsaturated fat, folate, vitamin D and vitamin E, and iron (women only). Yet again, these nutrients related to the text matching of breakfast cereals and baking and frying fats. The inclusion of these data illustrates the effect of text matching on the ranking of individuals for certain nutrients and will enable future researchers using FETA to make informed decisions on the benefit of text matching for their study.

We have not addressed or discussed common FFQ issues, such as the number of items in a food list or the use of a single average portion size, as these are not the focus of this paper and have been reviewed previously (24,25).

It is anticipated that future updates of FETA might contain a number of improvements and overcome some of the limitations of FETA, currently released as version 2.53 for Windows and Linux (last updated 15/03/2013 and 21/02/2013 respectively). The source code has been made available online which enables users to make modifications and improvements to the program. Currently, we have made available Windows and Linux versions and it is hoped that an OS X version will follow soon. We are currently working on a Libreoffice version of the Microsoft Access form-based entry tool.

In conclusion, we have created a new, open source, standalone, cross-platform FFQ processing tool, FETA, to produce nutrient and food group data for researchers using the EPIC-Norfolk FFQ. The tool produces similar nutrient and food group values to the previously validated CAFÉ program, but is more accessible. Although FETA was designed and based on the EPIC-Norfolk FFQ, the program was created in such a way that it can be customised for different study populations. It is anticipated that the development and availability of FETA will be a useful addition to the field of nutritional epidemiology and dietary public health.

Acknowledgments

For peer review only

We thank Mr Adam Dickinson, senior data manager at the MRC Epidemiology Unit, and his team members for their contribution to project management of FETA; Professor Nick Wareham, as EPIC-Norfolk study PI; and Mr Jamal Natour, as FETA software developer. The authors would also like to thank all the participants of the EPIC-Norfolk study and the EPIC-Norfolk staff for their help with this work.

Contributors

AAM contributed to the software development, assisted in statistical analyses and drafted the manuscript. AB and RL contributed to the software development, assisted in statistical analyses and contributed to the manuscript. DJP-S wrote the step-based graphical wizard for running FETA and contributed to the manuscript. NGF, LO'C and K-TK (Principal Investigator of EPIC-Norfolk) contributed to the manuscript. APK created the Microsoft Access form-based entry tool and contributed to the manuscript. All authors approved the final manuscript.

Funding

This study was supported by programme grants from the MRC Population Health Sciences Research Network (PHSRN), Cancer Research UK (C864/A8257) and the Medical Research Council (G0401527 and G1000143); NGF was supported by the Medical Research Council (MC_UP_A100_1003); APK is funded by a Wellcome Trust Clinical Research Fellowship.

Competing interests None.

Ethics approval Norwich Local Research Ethics Committee.

References

1. Subar AF. Developing dietary assessment tools. J Am Diet Assoc 2004 May;**104**(5):769–70. www.ncbi.nlm.nih.gov/pubmed/15127062 (accessed 5 Nov 2013).

For peer review only

2. Welch AA, Luben R, Khaw KT, et al. The CAFE computer program for nutritional analysis of the EPIC-Norfolk food frequency questionnaire and identification of extreme nutrient values. J Hum Nutr Diet 2005;**18**(2):99–116.

3. Bingham SA, Gill C, Welch A, et al. Validation of dietary assessment methods in the UK arm of EPIC using weighed records, and 24-hour urinary nitrogen and potassium and serum vitamin C and carotenoids as biomarkers. Int J Epidemiol 1997;**26**(1):S137–S151.

4. Bingham SA, Welch AA, McTaggart A, et al. Nutritional methods in the European Prospective Investigation of Cancer in Norfolk. Public Health Nutr 2001;**4**(3):847–58.

5. McKeown NM, Day NE, Welch AA, et al. Use of biological markers to validate self-reported dietary intake in a random sample of the European Prospective Investigation into Cancer United Kingdom Norfolk cohort. Am J Clin Nutr 2001;**74**:188–96.

6. Rimm EB, Giovannucci EL, Stampfer MJ, et al. Reproducibility and validity of an expanded self-administered semiquantitative food frequency questionnaire among male health professionals. Am J Epidemiol 1992 May;**135**(10):1114–26. www.ncbi.nlm.nih.gov/pubmed/1632423 (accessed 5 Nov 2013).

7. Bingham SA, Gill C, Welch A, et al. Comparison of dietary assessment methods in nutritional epidemiology: weighed records v. 24 h recalls, food-frequency questionnaires and estimated-diet records. Br J Nutr 1994;**72**(4):619–43.

8. Day N, Oakes S, Luben R, et al. EPIC-Norfolk: study design and characteristics of the cohort. European Prospective Investigation of Cancer. Br J Cancer 1999 Jul;**80**(Suppl 1):95–103. www.ncbi.nlm.nih.gov/pubmed/10466767 (accessed 5 Nov 2013).

9. Gregory J, Foster K, Tyler H, Wiseman M. The Dietary and Nutritional Survey of British Adults. London: Her Majesty's Stationary Office (HMSO) 1990.

For peer review only

10. MAFF. The Dietary and Nutritional Survey of British Adults - Further Analysis. London: Her Majesty's Stationary Office (HMSO) 1994.

11. MAFF. Food Portion Sizes. 2nd ed. London: Her Majesty's Stationary Office (HMSO) 1993.

12. Holland B, Unwin I, Buss D. Cereals and cereal products. The third supplement to McCance & Widdowson's The Composition of Foods (4th Edition). Cambridge: RSC/MAFF 1988.

13. Holland B, Unwin I, Buss D. Milk products and eggs. The fourth supplement to McCance & Widdowson's The Composition of Foods (4th Edition). Cambridge: RSC/MAFF 1989.

14. Holland B, Welch AA, Unwin D, Buss DH, Paul AA, Southgate DAT. McCance and Widdowson's The Composition of Foods. Cambridge: Royal Society of Chemistry (RSC) 1991.

15. Holland B, Unwin I, Buss D. Vegetables, herbs and spices. The fifth supplement to McCance & Widdowson's The Composition of Foods (4th Edition). Cambridge: RSC/MAFF 1991.

16. Holland B, Unwin I, Buss D. Fruit and nuts. The first supplement to McCance & Widdowson's The Composition of Foods (5th Edition). Cambridge: RSC/MAFF 1992.

17. Holland B, Welch A, Buss D. Vegetable dishes. The second supplement to McCance & Widdowson's The Composition of Foods (5th Edition). Cambridge: RSC/MAFF 1992.

18. Holland B, Brown J, Buss D. Fish and fish products. The third supplement to McCance & Widdowson's The Composition of Foods (5th Edition). Cambridge: RSC/MAFF 1993.

For peer review only

For peer review only - http://bmjopen.bmj.com/site/about/guidelines.xhtml

19. Chan W, Brown J, Buss D. Miscellaneous foods. The fourth supplement to McCance & Widdowson’s The Composition of Foods (5th Edition). Cambridge: RSC/MAFF 1994.

20. Chan W, Brown J, Lee S, Buss D. Meat, poultry and game. The fifth supplement to McCance & Widdowson’s The Composition of Foods (5th Edition). Cambridge: RSC/MAFF 1995.

21. Chan W, Brown J, Church S, Buss D. Meat products and dishes. The sixth supplement to McCance & Widdowson’s The Composition of Foods (5th Edition). Cambridge: RSC/MAFF 1996.

22. COMA. Dietary Reference Values for Food Energy and Nutrients for the United Kingdom. London: Her Majesty’s Stationary Office (HMSO) 1991.

23. Department of Health. www.nhs.uk/Livewell/5ADAY/Pages/5ADAYhome.aspx (accessed 5 Nov 2013).

24. Cade JE, Burley VJ, Warm DL, et al. Food-frequency questionnaires: a review of their design, validation and utilisation. Nutr Res Rev 2004 Jun;**17**(1):5–22. www.ncbi.nlm.nih.gov/pubmed/19079912 (accessed 30 Oct 2013).

25. Molag ML, de Vries JHM, Ocké MC, et al. Design characteristics of food frequency questionnaires in relation to their validity. Am J Epidemiol 2007 Dec;**166**(12):1468–78. www.ncbi.nlm.nih.gov/pubmed/17881382 (accessed 5 Nov 2013).

For peer review only

FOODS AND AMOUNTS	AVERAGE USE LAST YEAR								
**BREAD AND SAVOURY BISCUITS** (one slice or biscuit)	Never or less than once/month	1-3 per month	Once a week	2-4 per week	5-6 per week	Once a day	2-3 per day	4-5 per day	6+ per day
White bread and rolls						√			
Brown bread and rolls				√					
Wholemeal bread and rolls	√								
Cream crackers, cheese biscuits		√							
Crispbread, eg. Ryvita		√							
**CEREALS** (one bowl)									
Porridge, Readybrek				√					
Breakfast cereal such as cornflakes, muesli etc.					√				

Figure 1. Part 1 (main part) of the EPIC-Norfolk FFQ, illustrating bread, savoury biscuits and breakfast cereals

For peer review only

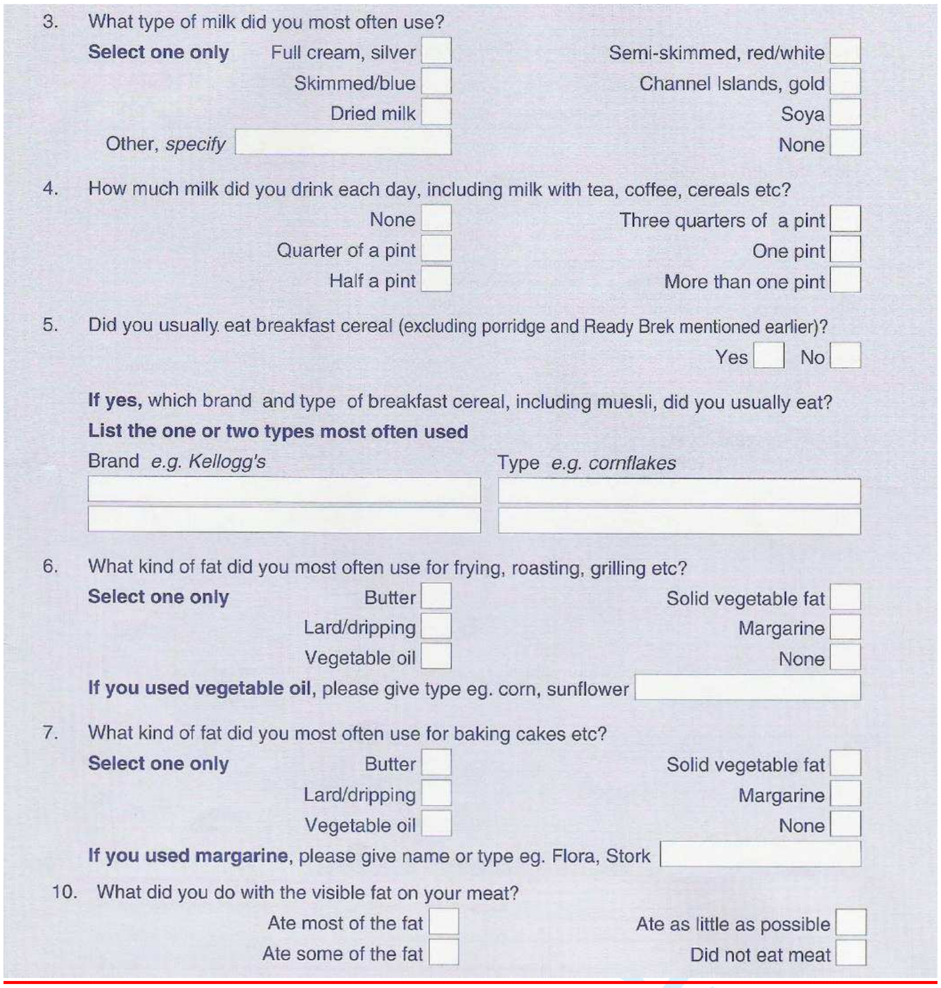

3. What type of milk did you most often use?

Select one only

Full cream, silver ☐ Semi-skimmed, red/white ☐
Skimmed/blue ☐ Channel Islands, gold ☐
Dried milk ☐ Soya ☐
Other, *specify* ☐ None ☐

4. How much milk did you drink each day, including milk with tea, coffee, cereals etc?

None ☐ Three quarters of a pint ☐
Quarter of a pint ☐ One pint ☐
Half a pint ☐ More than one pint ☐

5. Did you usually eat breakfast cereal (excluding porridge and Ready Brek mentioned earlier)?

Yes ☐ No ☐

If yes, which brand and type of breakfast cereal, including muesli, did you usually eat?

List the one or two types most often used

Brand *e.g. Kellogg's* Type *e.g. cornflakes*

6. What kind of fat did you most often use for frying, roasting, grilling etc?

Select one only

Butter ☐ Solid vegetable fat ☐
Lard/dripping ☐ Margarine ☐
Vegetable oil ☐ None ☐

If you used vegetable oil, please give type eg. corn, sunflower ☐

7. What kind of fat did you most often use for baking cakes etc?

Select one only

Butter ☐ Solid vegetable fat ☐
Lard/dripping ☐ Margarine ☐
Vegetable oil ☐ None ☐

If you used margarine, please give name or type eg. Flora, Stork ☐

10. What did you do with the visible fat on your meat?

Ate most of the fat ☐ Ate as little as possible ☐
Ate some of the fat ☐ Did not eat meat ☐

Figure 2. Questions from part 2 of the EPIC-Norfolk FFQ, used by FETA

w only

Appendix 1 Extract from a sample log file produced during the processing of 10 ids, using output 1.

2013-01-29 11:54 am: Note : Starting database setup

2013-01-29 11:54 am: Note : Loading imports for 'foods' completed

2013-01-29 11:54 am: Note : Loading imports for 'meals' completed

2013-01-29 11:54 am: Note : Loading imports for 'nutrients' completed

2013-01-29 11:54 am: Note : Loading imports for 'food_nutrients' completed

2013-01-29 11:54 am: Note : Loading imports for 'meal_foods' completed

2013-01-29 11:54 am: Note : Loading imports for 'weights' completed

2013-01-29 11:54 am: Note : Loading imports for 'portions' completed

2013-01-29 11:54 am: Note : Loading imports for 'frequencies' completed

2013-01-29 11:54 am: Note : Loading imports for 'cereals' completed

2013-01-29 11:54 am: Note : Loading imports for 'milks' completed

2013-01-29 11:54 am: Note : Completed database setup

2013-01-29 11:54 am: Error: Respondent: 001A supplied invalid frequency: -9 for meal: BURGER

2013-01-29 11:54 am: Error: Respondent: 001A supplied invalid frequency: -9 for meal: LIVER

2013-01-29 11:54 am: Error: Respondent: 003C supplied no baking fat food_codes

For peer review only

18 2013-01-29 11:54 am: Note : Respondent: 003C using default baking fat code: 17018

19 2013-01-29 11:54 am: Error: Respondent: 004D supplied invalid frequency: -9 for meal: FRUIT_SQUASH

20 2013-01-29 11:54 am: Error: Respondent: 005E supplied invalid frequency: -9 for meal: CHICKEN

21 2013-01-29 11:54 am: Error: Respondent: 005E supplied no frying fat food_codes

22 2013-01-29 11:54 am: Note : Respondent: 005E using default frying fat code: 17046

23 2013-01-29 11:54 am: Error: Respondent: 008H supplied invalid frequency: -9 for meal: INSTANT_COFFEE

24 2013-01-29 11:54 am: Error: Respondent: 008H supplied no baking fat food_codes

25 2013-01-29 11:54 am: Note : Respondent: 008H using default baking fat code: 17018

26 2013-01-29 11:54 am: Error: Respondent: 009J supplied invalid frequency: -9 for meal: DAIRY_DESSERT

27 2013-01-29 11:54 am: Error: Respondent: 009J supplied invalid frequency: -4 for meal: EGGS

28 2013-01-29 11:54 am: Error: Respondent: 009J supplied invalid frequency: -9 for meal: LOWCAL_SALAD_CREAM

29 2013-01-29 11:54 am: Error: Respondent: 009J supplied invalid frequency: -9 for meal: PLAIN_BISCUIT

30 2013-01-29 11:54 am: Error: Respondent: 009J supplied invalid frequency: -9 for meal: INSTANT_COFFEE

31 2013-01-29 11:54 am: Error: Respondent: 009J supplied invalid frequency: -9 for meal: COFFEE_WHITENER

32 2013-01-29 11:54 am: Error: Respondent: 009J supplied invalid frequency: -9 for meal: SPINACH

33 2013-01-29 11:54 am: Error: Respondent: 009J supplied no visible fat weighting

34 2013-01-29 11:54 am: Note : Respondent: 009J using default weighting: 1

For peer review only

2013-01-29 11:54 am: Error: Respondent: 010K supplied no visible fat weighting

2013-01-29 11:54 am: Note : Respondent: 010K using default weighting: 1

2013-01-29 11:54 am: Note : Processing completed for Respondent: 001A

2013-01-29 11:54 am: Note : Processing completed for Respondent: 002B

2013-01-29 11:54 am: Note : Processing completed for Respondent: 003C

2013-01-29 11:54 am: Note : Processing completed for Respondent: 004D

2013-01-29 11:54 am: Note : Processing completed for Respondent: 005E

2013-01-29 11:54 am: Note : Processing completed for Respondent: 006F

2013-01-29 11:54 am: Note : Processing completed for Respondent: 007G

2013-01-29 11:54 am: Note : Processing completed for Respondent: 008H

2013-01-29 11:54 am: Note : Processing completed for Respondent: 009J

2013-01-29 11:54 am: Note : Processing completed for Respondent: 010K

2013-01-29 11:54 am: Note : Questionaire: sample_input_290113.csv processing completed successfully, processed(10) respondents

For peer review only

A new tool for converting food frequency questionnaire data into nutrient and food group values: FETA research methods and availability

Journal:	*BMJ Open*
Manuscript ID:	bmjopen-2013-004503.R1
Article Type:	Research
Date Submitted by the Author:	04-Mar-2014

Complete List of Authors:	Mulligan, Angela; University of Cambridge, Public Health & Primary Care<br>Luben, Robert; University of Cambridge, Public Health & Primary Care<br>Bhaniani, Amit; University of Cambridge, Public Health & Primary Care<br>Parry-Smith, David; University of Cambridge, Public Health & Primary Care<br>O'Connor, Laura; University of Cambridge, Institute of Metabolic Science<br>Khawaja, Anthony; University of Cambridge, Public Health & Primary Care<br>Forouhi, Nita; University of Cambridge, Institute of Metabolic Science<br>Khaw, KayTee; University of Cambridge, Clinical Medicine

**Primary Subject Heading**:	Research methods
Secondary Subject Heading:	Health informatics, Public health, Nutrition and metabolism, Epidemiology
Keywords:	PUBLIC HEALTH, EPIDEMIOLOGY, Nutrient intakes, food frequency questionnaire

SCHOLARONE™
Manuscripts

For peer review only

A new tool for converting food frequency questionnaire data into nutrient and food group values: FETA research methods and availability

Angela A Mulligan[a1 c1], Robert N Luben[a1], Amit Bhaniani[a1], David J Parry-Smith[a1], Laura O'Connor[a2], Anthony P Khawaja[a1], Nita G Forouhi*[a2], Kay-Tee Khaw*[a1, a3]

* indicates equal contribution as authors

[a1] European Prospective Investigation into Cancer and Nutrition, Department of Public Health and Primary Care, University of Cambridge, Strangeways Research Laboratory, Worts Causeway, Cambridge, UK

[a2] MRC Epidemiology Unit, Institute of Metabolic Science, Addenbrooke's Hospital, University of Cambridge, Cambridge, UK

[a3] EPIC, Department of Gerontology, Addenbrooke's Hospital, School of Clinical Medicine, University of Cambridge, Cambridge, UK

For peer review only

Source of support: MRC Population Health Sciences Research Network (PHSRN), Cancer Research UK (C864/A8257) and the Medical Research Council (G0401527, G1000143)

Running title: FETA: new processing tool for FFQs

Key words: food frequency questionnaire, nutritional output, processing tool, EPIC-Norfolk

Correspondence:

[c1] Corresponding author: Ms A. Mulligan, telephone +44 1223 748683, fax +44 1223 748676, email angela.mulligan@phpc.cam.ac.uk

Abbreviations: FFQ, food frequency questionnaire; EPIC, European Prospective Investigation into Cancer and Nutrition; FETA, FFQ EPIC Tool for Analysis; CAFÉ, Compositional Analyses from Frequency Estimates

Word count: 3 380

ABSTRACT

Objectives

To describe the research methods for the development of a new open source, cross-platform tool which processes data from the European Prospective Investigation into Cancer and Nutrition Norfolk Food Frequency Questionnaire (EPIC-Norfolk FFQ). A further aim was to compare nutrient and food group values derived from the current tool (FETA; FFQ EPIC Tool for Analysis) with the previously validated but less accessible tool, CAFÉ (Compositional Analyses from Frequency Estimates). The effect of text matching on intake data was also investigated.

Design

Cross-sectional analysis of a prospective cohort study – EPIC-Norfolk.

Setting

East England population (city of Norwich and its surrounding small towns and rural areas).

Participants

Complete FFQ data from 11 250 men and 13 602 women (mean age 59 years; range 40 – 79 years).

Outcome measures

Nutrient and food group intakes derived from FETA and CAFÉ analyses of EPIC-Norfolk FFQ data.

Results

Nutrient outputs from FETA and CAFÉ were similar; mean (SD) energy intake from FETA was 9222 kJ (2633) in men, 8113 kJ (2296) in women, compared to CAFÉ intakes of 9175 kJ (2630) in men, 8091 kJ (2298) in women. The majority of differences resulted in one or less quintile change (98.7%). Only mean daily fruit and vegetable food group intakes were higher in women than in men (278 v 212 g and 284 v 255 g respectively). Quintile changes were evident for all nutrients, with the exception of alcohol, when text matching was not executed; however, only the cereals food group was affected.

Conclusions

For peer review only

FETA produces similar nutrient and food group values to the previously validated CAFÉ but has the advantages of being open source, cross-platform and complete with a data-entry form directly compatible with the software. The tool will facilitate research using the EPIC-Norfolk FFQ, and can be customised for different study populations.

Strengths and limitations of this study

- FETA has been tested using a large study sample of food intake data.
- No independent reference method used in the comparisons of FETA and CAFÉ nutrient intake data although the CAFÉ system has been previously validated.

INTRODUCTION

Food Frequency Questionnaires (FFQs) are commonly used in epidemiological studies to assess the dietary intake of large populations. Their popularity derives from ease of administration, ability to assess dietary intake over a defined period of time, and low costs (1). The European Prospective Investigation into Cancer and Nutrition (EPIC)-Norfolk FFQ is semi-quantitative and designed to record the average intake of foods during the previous year. The principles involved in data collection and processing of the EPIC-Norfolk FFQ and the development of the structure and content of the CAFÉ program for calculating nutrient intakes have been published previously (2). The EPIC-Norfolk FFQ has been extensively validated and has been widely used (3);(4);(5). However, the programs used to process these FFQs, including CAFÉ, have not been easily accessible to end-users.

Our objectives were to develop a new, open source, cross-platform processing tool (FETA - FFQ EPIC Tool for Analysis) based on and building upon the earlier system, CAFÉ (2). The aim of this report was to describe the research methods of the development of FETA, and to compare nutrient output from the FETA and CAFÉ programs. Food group intake data from FETA has also been described as has the effect of free text matching on nutrient and food group intake data. Free text

For peer review only

matching refers to the assigning of an appropriate food code to hand-written text in the FFQ and will be further described in the methods section.

METHODS

EPIC-FFQ design

The questionnaire consists of two parts. Part 1 consists of a food list of 130 lines; each line has a portion size attached to it: medium serving, standard unit or household measure. Study participants were requested to select an appropriate frequency of consumption for each line, from the nine frequency categories. As an example, Figure 1 illustrates the sections relating to bread, savoury biscuits and breakfast cereals. A pdf copy of the EPIC-Norfolk FFQ may be downloaded from http://www.srl.cam.ac.uk/epic/epicffq/websitedocumentation.html; information on how to complete and code the FFQ is also available here. The questionnaire lines are either individual foods, combinations of individual foods or food types. The FFQ food list is based on items from an FFQ widely used within the USA (6);(7), but modified to reflect differences in American versus UK brand names and some further food items were added.

Part 2 contains further questions, a number of which ask for more detailed information that link back to food lines in part one, as illustrated in Figure 2. Detailed information was requested for breakfast cereals and fats as these are nutritionally important foods in the UK diet.

Data collection

The EPIC-Norfolk FFQ was posted to 25 639 participants in the EPIC-Norfolk cohort study (8). The participants were aged 40-79 years and the questionnaire was completed between 1993 and 1997. The study was approved by the Norfolk Local Research Ethics Committee, adhered to the Declaration of Helsinki and all participants gave written informed consent. The FFQ was returned at a health examination, where it was checked and completed, if required, by trained nursing staff. In total, 25 351 (99%) participants returned the completed questionnaire.

Comparison of FETA and CAFÉ programs

For peer review only

FETA uses a csv (comma-separated values) input file. Part 1 is coded as numeric values and Part 2 is coded as numeric values and food codes, using the flow-charts and look-up lists provided (http://www.srl.cam.ac.uk/epic/epicffq/). We have also created a Microsoft Access form-based entry tool to facilitate FFQ data entry, based on the EPIC-Norfolk FFQ. The tool exports data in a format directly compatible with FETA. The FETA software was written in C and C++ languages, enabling faster processing times than SAS and the C/C++ software can also be used from the command line. The step-based graphical wizard for running FETA was written in Perl. Whereas in the CAFÉ program, an Oracle (Oracle Corporation, Redwood Shores, CA, USA) -based entry system was created to enter Part 1 frequency data as numeric codes and Part 2 data as numeric codes and free text. CAFÉ was written using SAS (SAS Software, Version 8 of the SAS System for Unix, SAS Institute Inc., Cary, NC, USA) and links to tables in an Oracle relational database.

Part 1- data entry

Data were manually entered into a spreadsheet as numeric codes, using ‘1’ for ‘never or less than once a month’, to ‘9’ for ‘6+ times per day’. A code of ‘-9’ was used to mark data where a frequency was not recorded. Where two frequencies were provided for a line, this was coded as ‘-4’ and treated by both CAFÉ and FETA programs as missing data. However, in FETA, both frequencies may now be entered, separated by a semi-colon, e.g. ‘2;3’, and FETA will process the first value.

Part 2 – assigning of food codes to ticked boxes and free text

Part 2 contains hand-written text for milk, breakfast cereals and cooking fats (see Figure 2, questions 3, 5, 6 and 7 respectively), which needs to be matched to the most appropriate food code in order to obtain nutrient data; this process is known as free text matching. The data in part 2 were coded using reference lists of food codes for varieties of milk, breakfast cereal and cooking fat. Where there is no clear match, it is suggested that a researcher consults the ingredients and nutrient information of the commercial item and compares this information with the nutrient profile of similar items from the reference lists. These reference lists and figures relating to food codes that

For peer review only

may be assigned to appropriate ticked boxes may be found at http://www.srl.cam.ac.uk/epic/epicffq/websitedocumentation.html Differences between FETA versus CAFÉ processing may also be found at http://www.srl.cam.ac.uk/epic/epicffq/websitedocumentation.html; these differences relate to breakfast cereals, frying and baking fats, the outcome of selecting the 'None' or 'No' box, and default milk, cereal, and fat codes.

Databases

Each line in Part 1 of the FFQ is mapped to up to six food codes. Decisions regarding which food codes to use were based on data from UK government surveys and other UK population data (7); (7,9,10). These decisions were based on data for individuals aged 40-74 years (7). Data for portion weights were sourced from UK population data and weighed records in 40-74 year old study participants (7,11).

The EPIC-Norfolk FFQ uses 290 foods from the UK food composition database, McCance and Widdowson's "The Composition of Foods" (5th edition) and its associated supplements (12–21). A number of new food items were added to the EPIC-Norfolk FFQ food list, which are used in both the FETA and CAFÉ programs. These include low calorie/diet fizzy drinks and crunchy oat cereal, as well as modified home-baked and fried foods (without their fat), to enable an individual's fat type, as recorded in Part 2 of the FFQ, to be incorporated. However, the nutrient data of six of the nine new foods used in the CAFÉ program were modified in FETA. These foods include crunchy oat cereal, milk non-specific, low calorie/diet fizzy drinks, solid vegetable oil, Crisp 'n Dry (solid fat), and oil and fat non-specific. Modifications to the nutrient data were made to ensure a more accurate nutrient profile and/or to better reflect the foods consumed, in the case of non-specific items, such as milk and oil/fat; these changes relate to nutrient/food data at the time of FFQ completion

Identification of outliers

For peer review only

Outliers were defined, as detailed previously (2). In brief, the ratio of energy intake (EI) to basal metabolic rate (BMR) was calculated, where BMR was calculated using sex-specific Schofield equations, which included age and body weight (22). Individuals in the top and bottom 0.5% of EI: BMR ratio were identified and excluded, as were individuals with FFQs containing 10 or more missing lines of data in Part 1of the FFQ.

Nutrient and food group outputs

FETA produces four nutrient output formats and a sample of each of these can be viewed at http://www.srl.cam.ac.uk/epic/epicffq/websitedocumentation.html

Output 1 contains average daily nutrient and food group intakes for an individual from all FFQ foods consumed, in wide format, suitable for import into a spreadsheet or statistical package. Intake data for 46 nutrients are provided as well as data for 14 basic food groups, however only a selection of these nutrients is shown in this report. Output 2 contains the same nutrient intake data as output 1, but in long format, which is mostly suitable for programmers. Output 3 contains average daily nutrient and food group intakes (and amount of food consumed) for an individual for each FFQ line; this output file will be very large and is mostly suitable for programmers. The most detailed output (output 4) contains average daily nutrient and food group intakes, in addition to the amount of food consumed for an individual, for each food code, for each FFQ line (meal_id). An online description of each meal_id and nutrient code, including units of measurement, can be found in the data entry template. This output will also be very large and is mostly suitable for programmers.

A log file is created along with each output file, which records the processing of the data and provides useful error information (see Appendix 1 for log file of output 1). In these files, both notes (general process information) and error messages are recorded, with a date and time stamp. The log files make it possible to calculate the number of missing frequencies based on Part 1 (main grid) of the FFQ in order to exclude individuals with 10 or more missing ticks. The log files also record situations where a food code does not have any nutrient data attached to it.

Statistical analyses

For peer review only

The data were analysed using STATA 10 (STATA Corp., Texas, USA). Intake data were described using mean, standard deviation (SD), median, minimum and maximum for both FETA and CAFÉ program outputs, stratified by sex. The nutrients selected for comparison are those described in the original CAFÉ paper. Where data on quintile changes are shown, cut-off points were calculated using CAFÉ nutrient data in order to compare quintile shift between FETA and CAFÉ output data.

RESULTS

We received FFQs from 25 351 participants (11 451 men and 13 900 women), with a mean age of 59 years. From this set, 249 FFQs (90 men and 159 women) containing 10 or more missing lines of data in Part 1 of the FFQ were excluded, followed by a further exclusion of 250 FFQs (111 men and 139 women) from the top and bottom 0.5% of EI:BMR. This resulted in the final analytical dataset of 24 852 participants (11 250 men and 13 602 women).

Nutrient intake data from FETA and CAFÉ programs

Table 1 shows the average daily intake data for a number of selected nutrients for 11 250 men. The data were similar for most nutrients across the two programs. The nutrients which had the highest percentage of quintile change (≥10%) were monounsaturated fat, saturated fat, iron, vitamin D & vitamin E. However, only 1.3% of the men changed more than one quintile, for two of these five nutrients. The nutrients which had the lowest percentage of quintile changes were alcohol, calcium and carotene, with less than 3% change (Table 1).

Table 2 shows average daily intake data for the selected nutrients for 13 602 women, from FETA and CAFÉ programs. There were similar quintile changes observed in women to those found in men for the selected nutrients; four of the nineteen nutrients had a quintile change of greater than 10%: polyunsaturated fat, saturated fat, iron and Vitamin E. However, the number of women who shifted more than one quintile was generally lower than the number observed in men. The nutrients which had the greatest percentage of women who changed more than one quintile were vitamins D and E, with 0.7 and 0.9% respectively.

For peer review only

Detailed (output 4) nutrient intake data at the individual level obtained from the two programs were compared for approximately half of the participants (n=12 500; data not shown). All differences (> 0.1%) found were investigated and explanations for these differences are considered in the discussion.

For peer review only

Table 1 Average daily nutrient intakes for men (N=11 250) participating in the EPIC-Norfolk study, from the FETA and CAFÉ programs, after the exclusion of outliers, with numbers and percentages of men who moved quintile

	FETA program					CAFÉ program								
Nutrient	Median	Mean	SD	Minimum	Maximum	Median	Mean	SD	Minimum	Maximum	Quintile change		Quintile change > 1	
											N	%	N	%
Energy (kcals)	2126	2190	627	748	5085	2115	2179	626	748	5101	892	7.9	0	0.0
Energy (kJs)	8947	9222	2633	3124	21394	8900	9175	2630	3124	21440	891	7.9	0	0.0
Protein (g)	83.4	85.2	22.0	23.3	319.8	83.2	84.9	22.0	23.3	318.4	464	4.1	0	0.0
Alcohol (g)	6.7	12.3	16.1	0.0	134.2	6.7	12.3	16.1	0.0	134.2	0	0.0	0	0.0
Carbohydrate (g)	261	271	87	48	737	259	269	87	48	729	726	6.5	0	0.0
Starch (g)	123	128	45	10	504	122	127	45	10	501	813	7.2	1	0.0
Englyst fibre (g)	17.5	18.2	6.4	1.3	89.9	17.3	18.0	6.4	1.3	89.9	743	6.6	1	0.0
Fat (g)	78.9	83.2	31.3	13.4	260.6	78.7	83.0	31.3	13.4	260.6	1049	9.3	8	0.1
Monounsaturated fat (g)	27.0	28.8	11.6	4.8	101.2	26.8	28.5	11.5	4.8	105.1	1264	11.2	21	0.2
Polyunsaturated fat (g)	13.5	15.0	6.9	1.6	66.6	13.7	15.3	7.1	1.6	69.5	1074	9.5	24	0.2
Saturated fat (g)	30.1	32.3	13.6	3.0	110.6	29.8	31.9	13.5	3.0	106.7	1288	11.5	20	0.2
Calcium (mg)	1021	1039	301	189	2848	1018	1037	300	189	2849	296	2.6	1	0.0
Iron (mg)	12.1	12.4	3.6	2.6	38.7	11.9	12.3	3.5	2.5	38.5	1149	10.2	7	0.1
Potassium (mg)	3814	3881	911	1305	11718	3802	3869	909	1284	11718	411	3.7	0	0.0

For peer review only

Carotene (mcg)	3188	3321	1573	147	25720	3178	3309	1571	147	25720	156	1.4	0	0.0
Folate (mcg)	320	331	97	77	1547	316	327	96	77	1547	836	7.4	3	0.0
Vitamin C (mg)	103	111	52	10	669	105	113	52	10	669	411	3.7	14	0.1
Vitamin D (mcg)	3.16	3.65	2.08	0.03	27.08	3.13	3.62	2.06	0.03	27.12	1161	10.3	145	1.3
Vitamin E (mg)	13.2	14.9	7.2	2.1	62.3	12.9	14.4	6.8	2.1	62.0	1545	13.7	146	1.3

For peer review only

Table 2 Average daily nutrient intakes for women (N=13 602) participating in the EPIC-Norfolk study, from the FETA and CAFÉ programs, after the exclusion of outliers, with numbers and percentages of women who moved quintile

	FETA program					CAFÉ program								
Nutrient	Median	Mean	SD	Minimum	Maximum	Median	Mean	SD	Minimum	Maximum	Quintile change		Quintile change > 1	
											N	%	N	%
Energy (kcals)	1859	1925	546	538	4733	1853	1920	547	518	4643	1030	7.6	0	0.0
Energy (kJs)	7833	8113	2296	2261	19910	7811	8091	2298	2179	19537	1018	7.5	0	0.0
Protein (g)	79.8	81.5	21.1	23.0	246.0	79.6	81.3	21.0	22.7	246.1	495	3.6	1	0.0
Alcohol (g)	2.0	5.6	8.4	0.0	99.5	2.0	5.6	8.4	0.0	99.5	0	0.0	0	0.0
Carbohydrate (g)	237	247	77	59	766	235	245	77	58	766	974	7.2	1	0.0
Starch (g)	107	112	39	13	405	106	111	39	13	406	1142	8.4	1	0.0
Englyst fibre (g)	18.2	19.0	6.8	2.3	118.5	18.0	18.8	6.7	2.4	118.6	850	6.2	1	0.0
Fat (g)	67.0	70.8	27.1	11.7	221.0	67.2	71.2	27.3	11.6	217.2	1194	8.8	4	0.0
Monounsaturated fat (g)	22.5	24.1	9.9	3.8	100.3	22.5	24.1	9.9	3.5	100.6	1338	9.8	7	0.1
Polyunsaturated fat (g)	12.2	13.5	6.2	2.0	53.6	12.5	13.8	6.3	2.0	53.6	1434	10.5	23	0.2
Saturated fat (g)	25.0	27.0	11.7	3.6	102.3	25.0	26.9	11.7	3.7	99.3	1443	10.6	9	0.1
Calcium (mg)	971	992	290	128	3159	969	990	290	127	3159	390	2.9	4	0.0
Iron (mg)	11.5	11.8	3.6	1.7	66.1	11.3	11.7	3.5	1.8	65.7	1496	11.0	12	0.1
Potassium (mg)	3781	3861	942	1150	16568	3769	3848	939	1147	16587	486	3.6	1	0.0
Carotene (mcg)	3477	3719	1917	67	61971	3469	3712	1917	64	61983	122	0.9	0	0.0

For peer review only

Folate (mcg)	322	332	103	65	2039	317	328	101	65	2024	1025	7.5	5	0.0
Vitamin C (mg)	123	133	64	4	1006	125	135	64	4	1006	746	5.5	35	0.3
Vitamin D (mcg)	3.01	3.46	1.90	0.00	17.83	3.02	3.45	1.90	0.00	17.75	1119	8.2	90	0.7
Vitamin E (mg)	12.4	13.8	6.2	1.5	52.4	12.2	13.5	6.0	1.6	49.8	1863	13.7	123	0.9

For peer review only

Food group intake data from FETA

Average daily intakes for both men and women of the fourteen food groups readily available from FETA are shown in Table 3. Mean daily intakes of six of the food groups were higher in men than in women: alcohol, cereals, fats, meat, potatoes and sugars. However, women had higher intakes of fruit (278g v 212g) and vegetables (284g v 255g). Mean daily intakes of eggs, fish, milk, non-alcoholic beverages, nuts and seeds, and soups and sauces were similar in both men and women.

The effect of text matching in FETA

Tables 4 and 5 illustrate the variation in nutrient and food group intake data obtained in a random subset of 1 159 men and 1 340 women, respectively, depending on whether text matching of milks, breakfast cereals and baking and frying fats was applied. In general, mean nutrient intakes were higher when text matching was carried out. In men, (Table 4), quintile changes (>15%) were most evident in the following nutrients: Englyst fibre, polyunsaturated fat, folate, vitamin D and vitamin E. The food group "cereals and cereal products" was the only one of the fourteen groups where there was a difference, with 31 men moving 1 quintile.

In women, (Table 5), quintile changes (>15%) were also most evident in the same five nutrients. However, almost 21% of women also changed quintile for iron. Once again, the "cereals and cereal products" food group was the only food group where there was any difference, with 40 women moving 1 quintile.

For peer review only

Table 3 Average daily food group intakes for men (N=11 250) and women (N=13 602) participating in the EPIC-Norfolk study, from the FETA program

	Men					Women				
Food group	Median	Mean	SD	Minimum	Maximum	Median	Mean	SD	Minimum	Maximum
Alcoholic beverages (g)	101	204	315	0	2483	23	64	109	0	1728
Cereals & cereal products (g)	242	260	127	0	1456	215	231	110	0	1172
Eggs & egg dishes (g)	18	17	15	0	225	14	16	14	0	236
Fats & oils (g)	31	36	22	0	207	27	30	20	0	218
Fish & fish products (g)	32	37	26	0	362	32	38	26	0	309
Fruit (g)	179	212	164	0	2654	238	278	201	0	3742
Meat & meat products (g)	99	106	54	0	856	91	94	48	0	606
Milk & milk products (g)	407	420	182	0	1303	386	410	175	0	1560
Non-alcoholic beverages (g)	1157	1177	396	0	3707	1150	1165	403	0	4501
Nuts & seeds (g)	0	3	9	0	228	0	3	9	0	188
Potatoes (g)	125	122	69	0	1007	116	112	64	0	1506
Soups & sauces (g)	43	58	54	0	1004	43	57	53	0	1376
Sugars (g)	53	64	50	0	572	37	48	42	0	541
Vegetables (g)	236	255	123	0	2398	262	284	143	0	3539

For peer review only

Table 4 Comparison of average daily nutrient and food group intakes for men (N=1 159) participating in the EPIC-Norfolk study, from the FETA program, with and without the application of text matching

Nutrient/Food group	FETA program, with text matching					FETA program, without text matching					Quintile change		Quintile change > 1	
	Median	Mean	SD	Minimum	Maximum	Median	Mean	SD	Minimum	Maximum	N	%	N	%
Energy (kcals)	2095	2176	678	658	7766	2091	2170	678	658	7787	28	2.4	0	0.0
Energy (kJs)	8822	9161	2848	2780	32555	8804	9138	2850	2780	32647	26	2.2	0	0.0
Protein (g)	82.8	85.0	22.8	22.1	272.3	82.5	84.7	22.8	22.1	272.3	34	2.9	0	0.0
Alcohol (g)	7.2	12.3	16.1	0.0	112.9	7.2	12.3	16.1	0.0	112.9	0	0.0	0	0.0
Carbohydrate (g)	261	270	93	63	1006	259	269	93	63	1003	48	4.1	0	0.0
Starch (g)	120	127	49	7	643	121	126	48	7	636	65	5.6	0	0.0
Englyst fibre (g)	17.5	18.3	6.6	3.6	71.8	17.3	17.9	6.3	3.6	64.5	198	17.1	10	0.9
Fat (g)	77.8	82.1	33.1	12.8	387.8	77.3	82.1	33.1	12.8	389.3	32	2.8	0	0.0
Monounsaturated fat (g)	26.5	28.2	12.2	3.5	131.1	26.7	28.7	12.5	3.7	138.7	88	7.6	0	0.0
Polyunsaturated fat (g)	13.5	14.9	7.3	3.0	67.0	12.7	14.1	6.8	3.0	60.7	179	15.4	17	1.5
Saturated fat (g)	30.1	31.8	14.1	3.3	160.0	30.3	32.2	14.3	3.3	160.3	72	6.2	1	0.1
Calcium (mg)	1015	1044	312	242	2848	1012	1044	313	242	2861	42	3.6	0	0.0
Iron (mg)	11.9	12.5	3.8	2.6	37.9	11.7	12.0	3.5	2.6	38.1	173	14.9	16	1.4
Potassium (mg)	3824	3889	957	1353	12675	3812	3873	951	1353	12551	52	4.5	0	0.0

For peer review only

For peer review only - http://bmjopen.bmj.com/site/about/guidelines.xhtml

Carotene (mcg)	3150	3348	1671	507	18295	3162	3353	1672	507	18338	6	0.5	0	0.0
Folate (mcg)	325	333	103	94	1222	316	326	101	94	1262	226	19.5	2	0.2
Vitamin C (mg)	105	113	55	17	619	104	112	55	17	619	22	1.9	0	0.0
Vitamin D (mcg)	3.08	3.64	2.17	0.03	16.40	3.06	3.64	2.19	0.03	20.52	227	19.6	8	0.7
Vitamin E (mg)	13.3	15.0	7.6	2.7	74.7	13.0	14.5	7.1	2.7	71.2	238	20.5	30	2.6
Alcoholic beverages (g)	104	201	301	0	1866	104	201	301	0	1866	0	0.0	0	0.0
Cereals & cereal products (g)	240	257	131	0	1378	238	255	130	0	1378	31	2.7	0	0.0
Eggs & egg dishes (g)	18	17	17	0	225	18	17	17	0	225	0	0.0	0	0.0
Fats & oils (g)	31	36	25	0	313	31	36	25	0	313	0	0.0	0	0.0
Fish & fish products (g)	32	37	25	0	153	32	37	25	0	153	0	0.0	0	0.0
Fruit (g)	184	216	158	0	1037	184	216	158	0	1037	0	0.0	0	0.0
Meat & meat products (g)	98	104	52	0	690	98	104	52	0	690	0	0.0	0	0.0
Milk & milk products (g)	414	428	187	0	1302	414	428	187	0	1302	0	0.0	0	0.0
Non-alcoholic beverages (g)	1159	1191	397	22	3677	1159	1191	397	22	3677	0	0.0	0	0.0
Nuts & seeds (g)	0	3	8	0	135	0	3	8	0	135	0	0.0	0	0.0
Potatoes (g)	125	121	78	0	1518	125	121	78	0	1518	0	0.0	0	0.0
Soups & sauces (g)	43	56	51	0	556	43	56	51	0	556	0	0.0	0	0.0
Sugars (g)	51	63	50	0	358	51	63	50	0	358	0	0.0	0	0.0
Vegetables (g)	238	256	128	15	1047	238	256	128	15	1047	0	0.0	0	0.0

For peer review only

Table 5 Comparison of average daily nutrient and food group intakes for women (N=1 340) participating in the EPIC-Norfolk study, from the FETA program, with and without the application of text matching

Nutrient/Food group	**FETA program, with text matching**					**FETA program, without text matching**								
	Median	Mean	SD	Minimum	Maximum	Median	Mean	SD	Minimum	Maximum	Quintile change		Quintile change > 1	
											N	%	N	%
Energy (kcals)	1886	1946	607	608	8103	1880	1941	605	608	8134	50	3.7	0	0.0
Energy (kJs)	7938	8202	2554	2552	34410	7909	8177	2547	2552	34541	47	3.5	0	0.0
Protein (g)	80.3	82.5	22.2	26.8	277.0	79.9	82.1	22.1	26.8	276.6	43	3.2	0	0.0
Alcohol (g)	2.0	5.4	8.1	0.0	65.3	2.0	5.4	8.1	0.0	65.3	0	0.0	0	0.0
Carbohydrate (g)	238	250	90	67	1596	237	249	90	67	1603	58	4.3	0	0.0
Starch (g)	109	114	52	25	1288	108	114	52	25	1301	99	7.4	0	0.0
Englyst fibre (g)	18.6	19.3	7.4	4.1	103.7	17.8	18.7	7.1	3.3	97.2	247	18.4	13	1.0
Fat (g)	67.6	71.4	28.5	17.2	259.4	67.5	71.3	28.4	17.2	259.7	45	3.4	0	0.0
Monounsaturated fat (g)	22.7	24.4	10.6	4.8	104.2	23.1	24.6	10.6	4.8	103.8	133	9.9	0	0.0
Polyunsaturated fat (g)	12.2	13.6	6.2	2.6	42.5	11.5	12.9	5.9	2.5	39.4	224	16.7	11	0.8
Saturated fat (g)	25.2	27.2	12.4	5.1	109.6	25.5	27.5	12.4	5.1	109.6	74	5.5	2	0.1
Calcium (mg)	978	995	298	242	2528	976	992	297	242	2534	46	3.4	1	0.1
Iron (mg)	11.7	11.9	3.9	3.1	67.8	11.1	11.4	3.5	3.1	55.3	280	20.9	44	3.3
Potassium (mg)	3788	3874	994	1284	12702	3744	3848	987	1280	12526	68	5.1	0	0.0

For peer review only

Carotene (mcg)	3489	3731	1705	178	13796	3500	3736	1707	175	13796	11	0.8	0	0.0
Folate (mcg)	326	337	107	102	1311	318	329	105	97	1276	291	21.7	1	0.1
Vitamin C (mg)	124	133	63	4	809	122	132	62	4	809	34	2.5	0	0.0
Vitamin D (mcg)	3.07	3.49	1.89	0.22	12.06	3.02	3.46	1.89	0.29	12.46	248	18.5	9	0.7
Vitamin E (mg)	12.5	13.8	6.3	2.7	52.4	12.1	13.3	5.9	3.3	43.6	270	20.2	21	1.6
Alcoholic beverages (g)	21	61	104	0	1350	21	61	104	0	1350	0	0.0	0	0.0
Cereals & cereal products (g)	214	236	174	9	4948	212	234	174	9	4948	40	3.0	0	0.0
Eggs & egg dishes (g)	14	16	14	0	136	14	16	14	0	136	0	0.0	0	0.0
Fats & oils (g)	27	30	19	0	133	27	30	19	0	133	0	0.0	0	0.0
Fish & fish products (g)	32	39	26	0	187	32	39	26	0	187	0	0.0	0	0.0
Fruit (g)	238	277	199	0	2830	238	277	199	0	2830	0	0.0	0	0.0
Meat & meat products (g)	90	95	49	0	392	90	95	49	0	392	0	0.0	0	0.0
Milk & milk products (g)	381	410	174	0	959	381	410	174	0	959	0	0.0	0	0.0
Non-alcoholic beverages (g)	1148	1153	404	8	3215	1148	1153	404	8	3215	0	0.0	0	0.0
Nuts & seeds (g)	0	3	11	0	180	0	3	11	0	180	0	0.0	0	0.0
Potatoes (g)	116	113	61	0	785	116	113	61	0	785	0	0.0	0	0.0
Soups & sauces (g)	45	57	53	0	900	45	57	53	0	900	0	0.0	0	0.0
Sugars (g)	38	50	46	0	540	38	50	46	0	540	0	0.0	0	0.0
Vegetables (g)	265	288	140	2	1387	265	288	140	2	1387	0	0.0	0	0.0

For peer review only

DISCUSSION

FETA provides a new, freely available, standalone tool that can produce nutrient and food group intake values from data collected using the EPIC-Norfolk FFQ. It makes the EPIC-Norfolk FFQ readily accessible to end-users and enables them to process and analyse nutritional data. The data can either be entered into a spreadsheet, using the instructions provided, or by using the specifically developed Microsoft Access form-based entry tool. The Access entry tool allows easier entry without requiring knowledge of specific food codes. The software for FETA for Windows and Linux can be downloaded from the website, as can the Microsoft Access data entry utility (http://www.srl.cam.ac.uk/epic/epicffq/). Users are encouraged to register with EPIC-Norfolk, as this enables them to request assistance and support. The various types of output (with four levels of information) available should prove beneficial to researchers, especially those requiring more detailed information. There is an on-going need for information on the intake of food groups. While the data from either output 3 or 4 could be used to generate more detailed food group data, we have treated food groups as another type of nutrient – a pseudo-nutrient. The FETA input/look-up files can be easily modified to create new groups, greatly adding to the flexibility of the system for analysing food group consumption, while requiring no spreadsheet or programming skills on the part of the analyst. A helpful feature of FETA is the log file which documents errors relating to FFQ data and/or default food codes assigned.

FETA was designed and based on the extensively validated EPIC-Norfolk FFQ, originally developed in 1988, to assess the nutrient and food group intake of 40-79 year olds, who completed the FFQ between 1993 and 1997. The food list and look-up lists of milks, breakfast cereals and fats reflect this time period and the study population, as do the default milk, cereal, baking fat and frying fat codes assigned. However, the program was created in such a way that it can be customised for different study populations, easily enabled by the separation of the processing algorithm in the FETA program implementation from the data model text files. It is possible to delete/add foods and/or FFQ lines, and modify portion sizes as desired for a study.

For peer review only

Nutrient data may also be easily modified or added. It is also possible for FETA to be used with other questionnaires containing a different set of line items or different numbers of frequencies. Comparisons were carried out for a number of selected nutrients obtained from FETA and the previously validated CAFÉ program. These showed that the nutrient output from both programs were generally similar. All differences (>0.1%) found from the comparison of detailed food/nutrient data at the individual level for 12 500 participants from FETA and the CAFÉ program can be explained by one or more of the following reasons: up to four cereal foods assigned by FETA, as compared to a maximum of two cereal foods assigned by CAFÉ; differences in default baking and frying fat codes assigned; correction for muesli portion size in cereal data; exclusion of porridge from cereal data (free text); default codes assigned for milk, cereals or fats to participants using FETA (where no food codes were assigned by CAFÉ program); rounding error (only where percentage absolute differences were between 0.1 to 1%) and changes made to the nutrient data of six of the nine new foods as well as to the default code for milk. A section entitled 'What are the differences between FETA versus CAFÉ processing?' found at http://www.srl.cam.ac.uk/epic/epicffq/FAQs.html further explains the aforementioned differences.

Although nutrient intakes as calculated by FETA and CAFÉ were similar, some relatively small differences existed, but these and the quintile shift of men and women can be explained. In FETA, a number of changes were made to the processing of breakfast cereals, affecting carbohydrate, starch, Englyst fibre, iron and folate estimates. The vitamin C content per 100g of low calorie/diet fizzy drinks was changed from 5 to 0 mg and the vitamin E content of crunchy oat cereal and oil and fat non-specific was increased. Changes made to the processing of fats in Questions 6 and 7 in Part 2 of the FFQ, in addition to changes made to the fatty acid profile of the three new fats, could help explain the small differences observed in monounsaturated, polyunsaturated and saturated fat intakes.

For peer review only

There was quite a large range in intake in the fourteen food groups, with a minimum intake of zero for each of the food groups. It is difficult to compare food group intake data as the groupings of foods often varies. However, the combined mean intake of fruit (excluding juices) and vegetables for men and women was 467g and 562g respectively, achieving the Government's 'Five a day' recommendation(23), using a portion size of 80 g.

Whilst text matching only affected one food group (cereals and cereal products), more than 15% of men and women changed quintile for a number of nutrients: Englyst fibre, polyunsaturated fat, folate, vitamin D and vitamin E, and iron (women only). Yet again, these nutrients related to the text matching of breakfast cereals and baking and frying fats. The inclusion of these data illustrates the effect of text matching on the ranking of individuals for certain nutrients and will enable future researchers using FETA to make informed decisions on the benefit of text matching for their study.

We have not addressed or discussed common FFQ issues, such as the number of items in a food list or the use of a single average portion size, as these are not the focus of this paper and have been reviewed previously (24,25).

It is anticipated that future updates of FETA might contain a number of improvements and overcome some of the limitations of FETA, currently released as version 2.53 for Windows and Linux (last updated 15/03/2013 and 21/02/2013 respectively). The source code has been made available online which enables users to make modifications and improvements to the program. Currently, we have made available Windows and Linux versions and it is hoped that an OS X version will follow soon. We are currently working on a Libreoffice version of the Microsoft Access form-based entry tool.

In conclusion, we have created a new, open source, standalone, cross-platform FFQ processing tool, FETA, to produce nutrient and food group data for researchers using the EPIC-Norfolk FFQ. The tool produces similar nutrient and food group values to the previously validated CAFÉ program, but is more accessible. Although FETA was designed and based on the EPIC-Norfolk

For peer review only

FFQ, the program was created in such a way that it can be customised for different study populations. It is anticipated that the development and availability of FETA will be a useful addition to the field of nutritional epidemiology and dietary public health.

For peer review only

Acknowledgments

We thank Mr Adam Dickinson, senior data manager at the MRC Epidemiology Unit, and his team members for their contribution to project management of FETA; Professor Nick Wareham, as EPIC-Norfolk study PI; and Mr Jamal Natour, as FETA software developer. The authors would also like to thank all the participants of the EPIC-Norfolk study and the EPIC-Norfolk staff for their help with this work.

Contributors

AAM contributed to the software development, assisted in statistical analyses and drafted the manuscript. AB and RL contributed to the software development, assisted in statistical analyses and contributed to the manuscript. DJP-S wrote the step-based graphical wizard for running FETA and contributed to the manuscript. NGF, LO'C and K-TK (Principal Investigator of EPIC-Norfolk) contributed to the manuscript. APK created the Microsoft Access form-based entry tool and contributed to the manuscript. All authors approved the final manuscript.

Funding

This study was supported by programme grants from the MRC Population Health Sciences Research Network (PHSRN), Cancer Research UK (C864/A8257) and the Medical Research Council (G0401527 and G1000143); NGF was supported by the Medical Research Council (MC_UP_A100_1003); APK is funded by a Wellcome Trust Clinical Research Fellowship.

Competing interests None.

Data Sharing Statement: Epic-Norfolk has a wide range of collaborators. Contact details, publications and the process for collaborating and data requests can be found on the website (www.epic-norfolk.org.uk).

Ethics approval Norwich Local Research Ethics Committee.

For peer review only

References

1. Subar AF. Developing dietary assessment tools. J Am Diet Assoc 2004 May;**104**(5):769–70. www.ncbi.nlm.nih.gov/pubmed/15127062 (accessed 5 Nov 2013).

2. Welch AA, Luben R, Khaw KT, et al. The CAFE computer program for nutritional analysis of the EPIC-Norfolk food frequency questionnaire and identification of extreme nutrient values. J Hum Nutr Diet 2005;**18**(2):99–116.

3. Bingham SA, Gill C, Welch A, et al. Validation of dietary assessment methods in the UK arm of EPIC using weighed records, and 24-hour urinary nitrogen and potassium and serum vitamin C and carotenoids as biomarkers. Int J Epidemiol 1997;**26**(1):S137–S151.

4. Bingham SA, Welch AA, McTaggart A, et al. Nutritional methods in the European Prospective Investigation of Cancer in Norfolk. Public Health Nutr 2001;**4**(3):847–58.

5. McKeown NM, Day NE, Welch AA, et al. Use of biological markers to validate self-reported dietary intake in a random sample of the European Prospective Investigation into Cancer United Kingdom Norfolk cohort. Am J Clin Nutr 2001;**74**:188–96.

6. Rimm EB, Giovannucci EL, Stampfer MJ, et al. Reproducibility and validity of an expanded self-administered semiquantitative food frequency questionnaire among male health professionals. Am J Epidemiol 1992 May;**135**(10):1114–26. www.ncbi.nlm.nih.gov/pubmed/1632423 (accessed 5 Nov 2013).

7. Bingham SA, Gill C, Welch A, et al. Comparison of dietary assessment methods in nutritional epidemiology: weighed records v. 24 h recalls, food-frequency questionnaires and estimated-diet records. Br J Nutr 1994;**72**(4):619–43.

For peer review only

8. Day N, Oakes S, Luben R, et al. EPIC-Norfolk: study design and characteristics of the cohort. European Prospective Investigation of Cancer. Br J Cancer 1999 Jul;**80**(Suppl 1):95–103. www.ncbi.nlm.nih.gov/pubmed/10466767 (accessed 5 Nov 2013).

9. Gregory J, Foster K, Tyler H, et al.. The Dietary and Nutritional Survey of British Adults. London: Her Majesty's Stationary Office (HMSO) 1990.

10. MAFF. The Dietary and Nutritional Survey of British Adults - Further Analysis. London: Her Majesty's Stationary Office (HMSO) 1994.

11. MAFF. Food Portion Sizes. 2nd ed. London: Her Majesty's Stationary Office (HMSO) 1993.

12. Holland B, Unwin I, Buss D. Cereals and cereal products. The third supplement to McCance & Widdowson's The Composition of Foods (4th Edition). Cambridge: RSC/MAFF 1988.

13. Holland B, Unwin I, Buss D. Milk products and eggs. The fourth supplement to McCance & Widdowson's The Composition of Foods (4th Edition). Cambridge: RSC/MAFF 1989.

14. Holland B, Welch AA, Unwin D, et al.. McCance and Widdowson's The Composition of Foods. Cambridge: Royal Society of Chemistry (RSC) 1991.

15. Holland B, Unwin I, Buss D. Vegetables, herbs and spices. The fifth supplement to McCance & Widdowson's The Composition of Foods (4th Edition). Cambridge: RSC/MAFF 1991.

16. Holland B, Unwin I, Buss D. Fruit and nuts. The first supplement to McCance & Widdowson's The Composition of Foods (5th Edition). Cambridge: RSC/MAFF 1992.

For peer review only

17. Holland B, Welch A, Buss D. Vegetable dishes. The second supplement to McCance & Widdowson's The Composition of Foods (5th Edition). Cambridge: RSC/MAFF 1992.

18. Holland B, Brown J, Buss D. Fish and fish products. The third supplement to McCance & Widdowson's The Composition of Foods (5th Edition). Cambridge: RSC/MAFF 1993.

19. Chan W, Brown J, Buss D. Miscellaneous foods. The fourth supplement to McCance & Widdowson's The Composition of Foods (5th Edition). Cambridge: RSC/MAFF 1994.

20. Chan W, Brown J, Lee S, et al.. Meat, poultry and game. The fifth supplement to McCance & Widdowson's The Composition of Foods (5th Edition). Cambridge: RSC/MAFF 1995.

21. Chan W, Brown J, Church S,et al. Meat products and dishes. The sixth supplement to McCance & Widdowson's The Composition of Foods (5th Edition). Cambridge: RSC/MAFF 1996.

22. COMA. Dietary Reference Values for Food Energy and Nutrients for the United Kingdom. London: Her Majesty's Stationary Office (HMSO) 1991.

23. Department of Health. www.nhs.uk/Livewell/5ADAY/Pages/5ADAYhome.aspx (accessed 5 Nov 2013).

24. Cade JE, Burley VJ, Warm DL, et al. Food-frequency questionnaires: a review of their design, validation and utilisation. Nutr Res Rev 2004 Jun;**17**(1):5–22. www.ncbi.nlm.nih.gov/pubmed/19079912 (accessed 30 Oct 2013).

25. Molag ML, de Vries JHM, Ocké MC, et al. Design characteristics of food frequency questionnaires in relation to their validity. Am J Epidemiol 2007 Dec;**166**(12):1468–78. www.ncbi.nlm.nih.gov/pubmed/17881382 (accessed 5 Nov 2013).

For peer review only

451 **Figure Legends**

452 **Figure 1:** Part 1 (main part) of the EPIC-Norfolk FFQ, illustrating bread, savoury biscuits and
453 breakfast cereals

454 **Figure 2:** Questions from part 2 of the EPIC-Norfolk FFQ, used by FETA

For peer review only

A new tool for converting food frequency questionnaire data into nutrient and food group values: FETA research methods and availability

Angela A Mulligan[a1 c1], Robert N Luben[a1], Amit Bhaniani[a1], David J Parry-Smith[a1], Laura O'Connor[a2], Anthony P Khawaja[a1], Nita G Forouhi*[a2], Kay-Tee Khaw*[a1, a3]

* indicates equal contribution as authors

[a1] European Prospective Investigation into Cancer and Nutrition, Department of Public Health and Primary Care, University of Cambridge, Strangeways Research Laboratory, Worts Causeway, Cambridge, UK

[a2] MRC Epidemiology Unit, Institute of Metabolic Science, Addenbrooke's Hospital, University of Cambridge, Cambridge, UK

[a3] EPIC, Department of Gerontology, Addenbrooke's Hospital, School of Clinical Medicine, University of Cambridge, Cambridge, UK

Source of support: MRC Population Health Sciences Research Network (PHSRN), Cancer Research UK (C864/A8257) and the Medical Research Council (G0401527, G1000143)

Running title: FETA: new processing tool for FFQs

Key words: food frequency questionnaire, nutritional output, processing tool, EPIC-Norfolk

Correspondence:

[c1] Corresponding author: Ms A. Mulligan, telephone +44 1223 748683, fax +44 1223 748676, email angela.mulligan@phpc.cam.ac.uk

Abbreviations: FFQ, food frequency questionnaire; EPIC, European Prospective Investigation into Cancer and Nutrition; FETA, FFQ EPIC Tool for Analysis; CAFÉ, Compositional Analyses from Frequency Estimates

Word count: 3 380~~254~~

For peer review only

ABSTRACT

Objectives

To describe the research methods for the development of a new open source, cross-platform tool which processes data from the European Prospective Investigation into Cancer and Nutrition Norfolk Food Frequency Questionnaire (EPIC-Norfolk FFQ). A further aim was to compare nutrient and food group values derived from the current tool (FETA; FFQ EPIC Tool for Analysis) with the previously validated but less accessible tool, CAFÉ (Compositional Analyses from Frequency Estimates). The effect of text matching on intake data was also investigated.

Design

Cross-sectional analysis of a prospective cohort study – EPIC-Norfolk.

Setting

East England population (city of Norwich and its surrounding small towns and rural areas).

Participants

Complete FFQ data from 11 250 men and 13 602 women ~~with a~~ (mean age ~~of~~ 59 years; ~~(~~range 40 – 79 years).

Outcome measures

Nutrient and food group intakes derived from FETA and CAFÉ analyses of EPIC-Norfolk FFQ data.

Results

Nutrient outputs from FETA and CAFÉ were similar; mean (SD) energy intake from FETA was 9222 kJ (2633) in men, 8113 kJ (2296) in women, compared to CAFÉ intakes of 9175 kJ (2630) in men, 8091 kJ (2298) in women. The majority of differences resulted in one or less quintile change (98.7%). Only mean daily fruit and vegetable food group intakes were higher in women than in men (278 v 212 g and 284 v 255 g respectively). Quintile changes were evident for all nutrients, with the exception of alcohol, when text matching was not executed; however, only the cereals food group was affected.

For peer review only

Conclusions

FETA produces similar nutrient and food group values to the previously validated CAFÉ but has the advantages of being open source, cross-platform and complete with a data-entry form directly compatible with the software. The tool will facilitate research using the EPIC-Norfolk FFQ, and can be customised for different study populations.

Strengths and limitations of this study

- FETA has been tested using a large study sample of food intake data.
- No independent reference method used in the comparisons of FETA~~eta~~ and CAFÉ nutrient intake data although the CAFÉ system has been previously validated.

INTRODUCTION

Food Frequency Questionnaires (FFQs) are commonly used in epidemiological studies to assess the dietary intake of large populations. Their popularity derives from ease of administration, ability to assess dietary intake over a defined period of time, and low costs (1). The European Prospective Investigation into Cancer and Nutrition (EPIC)-Norfolk FFQ is semi-quantitative and designed to record the average intake of foods during the previous year. The principles involved in data collection and processing of the EPIC-Norfolk FFQ and the development of the structure and content of the CAFÉ program for calculating nutrient intakes have been published previously (2). The EPIC-Norfolk FFQ has been extensively validated and has been widely used (3);(4);(5). However, the programs used to process these FFQs, including CAFÉ, have not been easily accessible to end-users.

Our objectives were to develop a new, open source, cross-platform processing tool (FETA - FFQ EPIC Tool for Analysis) based on and building upon the earlier system, CAFÉ (2). The aim of this report was to describe the research methods of the development of FETA, and to compare nutrient output from the FETA and CAFÉ programs. Food group intake data from FETA has also been described as has the effect of free text matching on nutrient and food group intake data. Free text

For peer review only

matching refers to the assigning of an appropriate food code to hand-written text in the FFQ and will be further described in the methods section.

METHODS

EPIC-FFQ design

The questionnaire consists of two parts. Part 1 consists of a food list of 130 lines; each line has a portion size attached to it: medium serving, standard unit or household measure. Study participants were requested to select an appropriate frequency of consumption for each line, from the nine frequency categories. As an example, Figure 1 illustrates the sections relating to bread, savoury biscuits and breakfast cereals. A pdf copy of the EPIC-Norfolk FFQ may be downloaded from http://www.srl.cam.ac.uk/epic/epicffq/websitedocumentation.html; information on how to complete and code the FFQ is also available here. The questionnaire lines are either individual foods, combinations of individual foods or food types. The FFQ food list is based on items from an FFQ widely used within the USA (6);(7), but modified to reflect differences in American versus UK brand names and some further food items were added.

Part 2 contains further questions, a number of which ask for more detailed information that link back to food lines in part one, as illustrated in Figure 2. Detailed information was requested for breakfast cereals and fats as these are nutritionally important foods in the UK diet.

Data collection

The EPIC-Norfolk FFQ was posted to 25 639 participants in the EPIC-Norfolk cohort study (8). The participants were aged 40-79 years and the questionnaire was completed between 1993 and 1997. The study was approved by the Norfolk Local Research Ethics Committee, adhered to the Declaration of Helsinki and all participants gave written informed consent. The FFQ was returned at a health examination, where it was checked and completed, if required, by trained nursing staff. In total, 25 351 (99%) participants returned the completed questionnaire.

Comparison of FETA and CAFÉ programs

For peer review only

FETA uses a csv (comma-separated values) input file. Part 1 is coded as numeric values and Part 2 is coded as numeric values and food codes, using the flow-charts and look-up lists provided (http://www.srl.cam.ac.uk/epic/epicffq/). We have also created a Microsoft Access form-based entry tool to facilitate FFQ data entry, based on the EPIC-Norfolk FFQ. The tool exports data in a format directly compatible with FETA. The FETA software was written in C and C++ languages, enabling faster processing times than SAS and the C/C++ software can also be used from the command line. The step-based graphical wizard for running FETA was written in Perl. Whereas in the CAFÉ program, an Oracle (Oracle Corporation, Redwood Shores, CA, USA) -based entry system was created to enter Part 1 frequency data as numeric codes and Part 2 data as numeric codes and free text. CAFÉ was written using SAS (SAS Software, Version 8 of the SAS System for Unix, SAS Institute Inc., Cary, NC, USA) and links to tables in an Oracle relational database.

Part 1- data entry

Data were manually entered into a spreadsheet as numeric codes, using '1' for 'never or less than once a month', to '9' for '6+ times per day'. A code of '-9' was used to mark data where a frequency was not recorded. Where two frequencies were provided for a line, this~~ese~~ was~~ere~~ coded as '-4' and treated by both CAFÉ and FETA programs as missing data. However, in FETA, both frequencies may now be entered~~both coded~~, separated by a semi-colon, e.g. '2;3', and FETA will process~~ed~~ the first value. ~~In the CAFÉ program, two entries per line were treated as missing data.~~

Part 2 – assigning of food codes to ticked boxes and free text

Part 2 contains hand-written text for milk, breakfast cereals and cooking fats (see Figure 2, questions 3, 5, 6 and 7 respectively), which needs to be matched to the most appropriate food code in order to obtain nutrient data; this process is known as free text matching. The data in part 2 were coded using reference lists of food codes for varieties of milk, breakfast cereal and cooking fat. Where there is no clear match, it is suggested that a researcher consults the ingredients and nutrient information of the commercial item and compares this information with the nutrient profile of similar items from the reference lists. These reference lists and figures relating to food codes that

For peer review only

may be assigned to appropriate ticked boxes may be found at http://www.srl.cam.ac.uk/epic/epicffq/websitedocumentation.html

Differences between FETA versus CAFÉ processing may also be found at http://www.srl.cam.ac.uk/epic/epicffq/websitedocumentation.html; these differences relate to breakfast cereals, frying and baking fats, the outcome of selecting the 'None' or 'No' box, and default milk, cereal, and fat codes.

Databases

Each line in Part 1 of the FFQ is mapped to up to six food codes. Decisions regarding which food codes to use were based on data from UK government surveys and other UK population data (7); (7,9,10). These decisions were based on data for individuals aged 40-74 years (7). Data for portion weights were sourced from UK population data and weighed records in 40-74 year old study participants (7,11).

The EPIC-Norfolk FFQ uses 290 foods from the UK food composition database, McCance and Widdowson's "The Composition of Foods" (5th edition) and its associated supplements (12–21). A number of new food items were added to the EPIC-Norfolk FFQ food list, which are used in both their~~in~~ FETA and CAFÉ programs. These include low calorie/diet fizzy drinks and crunchy oat cereal, as well as modified home-baked and fried foods (without their fat), to enable an individual's fat type, as recorded in Part 2 of the FFQ, to be incorporated. However, the nutrient data of six of the nine new foods used in the CAFÉ program were modified in FETA. These foods include crunchy oat cereal, milk non-specific, low calorie/diet fizzy drinks, solid vegetable oil, Crisp 'n Dry (solid fat), and oil and fat non-specific. Modifications to the nutrient data were made to ensure a more accurate nutrient profile and/or to better reflect the foods consumed,~~-~~ in the case of non-specific items, such as milk and oil/fat; these changes relate to nutrient/food data at the time of FFQ completion~~.~~

Identification of outliers

For peer review only

Outliers were defined, as detailed previously (2). In brief, the ratio of energy intake (EI) to basal metabolic rate (BMR) was calculated, where BMR was calculated using sex-specific Schofield equations, which included age and body weight (22). Individuals in the top and bottom 0.5% of EI: BMR ratio were identified and excluded, as were individuals with FFQs containing 10 or more missing lines of data in Part 1of the FFQ.

Nutrient and food group outputs

FETA produces four nutrient output formats and a sample of each of these can be viewed at http://www.srl.cam.ac.uk/epic/epicffq/websitedocumentation.html

Output 1 contains average daily nutrient and food group intakes for an individual from all FFQ foods consumed, in wide format, suitable for import into a spreadsheet or statistical package. Intake data for 46 nutrients are provided as well as data for 14 basic food groups, however only a selection of these nutrients is shown in this report. Output 2 contains the same nutrient intake data as output 1, but in long format, which is mostly suitable for programmers. Output 3 contains average daily nutrient and food group intakes (and amount of food consumed) for an individual for each FFQ line; this output file will be very large and is mostly suitable for programmers. The most detailed output (output 4) contains average daily nutrient and food group intakes, in addition to the amount of food consumed for an individual, for each food code, for each FFQ line (meal_id). An online description of each meal_id and nutrient code, including units of measurement, can be found in the data entry template. This output will also be very large and is mostly suitable for programmers.

A log file is created along with each output file, which records the processing of the data and provides useful error information (see Appendix 1 for log file of output 1). In these files, both notes (general process information) and error messages are recorded, with a date and time stamp. The log files make it possible to calculate the number of missing frequencies based on Part 1 (main grid) of the FFQ in order to exclude individuals with 10 or more missing ticks. The log files also record situations where a food code does not have any nutrient data attached to it.

Statistical analyses

For peer review only

The data were analysed using STATA 10 (STATA Corp., Texas, USA). Intake data were described using mean, standard deviation (SD), median, minimum and maximum for both FETA and CAFÉ program outputs, stratified by sex. The nutrients selected for comparison are those described in the original CAFÉ paper. Where data on quintile changes are shown, cut-off points were calculated using CAFÉ nutrient data in order to compare quintile shift between FETA and CAFÉ output data.

RESULTS

We received~~There were~~ FFQs~~ data available~~ from 25 351 participants (11 451 men and 13 900 women), with a mean age of 59 years. From this set, 249 FFQs (90 men and 159 women) containing 10 or more missing lines of data in Part 1 of the FFQ were excluded, followed by a further exclusion of 250 FFQs (111 men and 139 women) from the top and bottom 0.5% of EI:BMR. This resulted in the final analytical dataset of 24 852 participants ~~Data from~~ (11 250 men and 13 602 women). ~~are presented here, as individuals in the top and bottom 0.5% of EI: BMR ratio have been excluded, as have individuals with FFQs containing 10 or more missing lines of data in Part 1of the FFQ.~~

Nutrient intake data from FETA and CAFÉ programs

Table 1 shows the average daily intake data for a number of selected nutrients for 11 250 men. The data were similar for most nutrients across the two programs. The nutrients which had the highest percentage of quintile change (≥10%) were monounsaturated fat, saturated fat, iron, vitamin D & vitamin E. However, only 1.3% of the men changed more than one quintile, for two of these five nutrients. The nutrients which had the lowest percentage of quintile changes were alcohol, calcium and carotene, with less than 3% change (Table 1).

Table 2 shows average daily intake data for the selected nutrients for 13 602 women, from FETA and CAFÉ programs. There were similar quintile changes observed in women to those found in men for the selected nutrients; four of the nineteen nutrients had a quintile change of greater than 10%: polyunsaturated fat, saturated fat, iron and Vitamin E. However, the number of women who shifted

For peer review only

more than one quintile was generally lower than the number observed in men. The nutrients which had the greatest percentage of women who changed more than one quintile were vitamins D and E, with 0.7 and 0.9% respectively.

Detailed (output 4) nutrient intake data at the individual level obtained from the two programs were compared for approximately half of the participants (n=12 500; data not shown). All differences (> 0.1%) found were investigated and explanations for these differences are considered in the discussion.

For peer review only

Table 1 Average daily nutrient intakes for men (N=11 250) participating in the EPIC-Norfolk study, from the FETA and CAFÉ programs, after the exclusion of outliers, with numbers and percentages of men who moved quintile

	FETA program					CAFÉ program								
Nutrient	Median	Mean	SD	Minimum	Maximum	Median	Mean	SD	Minimum	Maximum	Quintile change		Quintile change > 1	
											N	%	N	%
Energy (kcals)	2126	2190	627	748	5085	2115	2179	626	748	5101	892	7.9	0	0.0
Energy (kJs)	8947	9222	2633	3124	21394	8900	9175	2630	3124	21440	891	7.9	0	0.0
Protein (g)	83.4	85.2	22.0	23.3	319.8	83.2	84.9	22.0	23.3	318.4	464	4.1	0	0.0
Alcohol (g)	6.7	12.3	16.1	0.0	134.2	6.7	12.3	16.1	0.0	134.2	0	0.0	0	0.0
Carbohydrate (g)	261	271	87	48	737	259	269	87	48	729	726	6.5	0	0.0
Starch (g)	123	128	45	10	504	122	127	45	10	501	813	7.2	1	0.0
Englyst fibre (g)	17.5	18.2	6.4	1.3	89.9	17.3	18.0	6.4	1.3	89.9	743	6.6	1	0.0
Fat (g)	78.9	83.2	31.3	13.4	260.6	78.7	83.0	31.3	13.4	260.6	1049	9.3	8	0.1
Monounsaturated fat (g)	27.0	28.8	11.6	4.8	101.2	26.8	28.5	11.5	4.8	105.1	1264	11.2	21	0.2
Polyunsaturated fat (g)	13.5	15.0	6.9	1.6	66.6	13.7	15.3	7.1	1.6	69.5	1074	9.5	24	0.2
Saturated fat (g)	30.1	32.3	13.6	3.0	110.6	29.8	31.9	13.5	3.0	106.7	1288	11.5	20	0.2
Calcium (mg)	1021	1039	301	189	2848	1018	1037	300	189	2849	296	2.6	1	0.0
Iron (mg)	12.1	12.4	3.6	2.6	38.7	11.9	12.3	3.5	2.5	38.5	1149	10.2	7	0.1
Potassium (mg)	3814	3881	911	1305	11718	3802	3869	909	1284	11718	411	3.7	0	0.0

Formatted Table

Formatted: Right

Formatted: Right

For peer review only

Carotene (mcg)	3188	3321	1573	147	25720	3178	3309	1571	147	25720	156	1.4	0	0.0
Folate (mcg)	320	331	97	77	1547	316	327	96	77	1547	836	7.4	3	0.0
Vitamin C (mg)	103	111	52	10	669	105	113	52	10	669	411	3.7	14	0.1
Vitamin D (mcg)	3.16	3.65	2.08	0.03	27.08	3.13	3.62	2.06	0.03	27.12	1161	10.3	145	1.3
Vitamin E (mg)	13.2	14.9	7.2	2.1	62.3	12.9	14.4	6.8	2.1	62.0	1545	13.7	146	1.3

For peer review only

Table 2 Average daily nutrient intakes for women (N=13 602) participating in the EPIC-Norfolk study, from the FETA and CAFÉ programs, after the exclusion of outliers, with numbers and percentages of women who moved quintile

	FETA program					CAFÉ program								
Nutrient	Median	Mean	SD	Minimum	Maximum	Median	Mean	SD	Minimum	Maximum	Quintile change		Quintile change > 1	
											N	%	N	%
Energy (kcals)	1859	1925	546	538	4733	1853	1920	547	518	4643	1030	7.6	0	0.0
Energy (kJs)	7833	8113	2296	2261	19910	7811	8091	2298	2179	19537	1018	7.5	0	0.0
Protein (g)	79.8	81.5	21.1	23.0	246.0	79.6	81.3	21.0	22.7	246.1	495	3.6	1	0.0
Alcohol (g)	2.0	5.6	8.4	0.0	99.5	2.0	5.6	8.4	0.0	99.5	0	0.0	0	0.0
Carbohydrate (g)	237	247	77	59	766	235	245	77	58	766	974	7.2	1	0.0
Starch (g)	107	112	39	13	405	106	111	39	13	406	1142	8.4	1	0.0
Englyst fibre (g)	18.2	19.0	6.8	2.3	118.5	18.0	18.8	6.7	2.4	118.6	850	6.2	1	0.0
Fat (g)	67.0	70.8	27.1	11.7	221.0	67.2	71.2	27.3	11.6	217.2	1194	8.8	4	0.0
Monounsaturated fat (g)	22.5	24.1	9.9	3.8	100.3	22.5	24.1	9.9	3.5	100.6	1338	9.8	7	0.1
Polyunsaturated fat (g)	12.2	13.5	6.2	2.0	53.6	12.5	13.8	6.3	2.0	53.6	1434	10.5	23	0.2
Saturated fat (g)	25.0	27.0	11.7	3.6	102.3	25.0	26.9	11.7	3.7	99.3	1443	10.6	9	0.1
Calcium (mg)	971	992	290	128	3159	969	990	290	127	3159	390	2.9	4	0.0
Iron (mg)	11.5	11.8	3.6	1.7	66.1	11.3	11.7	3.5	1.8	65.7	1496	11.0	12	0.1
Potassium (mg)	3781	3861	942	1150	16568	3769	3848	939	1147	16587	486	3.6	1	0.0
Carotene (mcg)	3477	3719	1917	67	61971	3469	3712	1917	64	61983	122	0.9	0	0.0

Formatted Table

Formatted: Right

Formatted: Right

For peer review only

Folate (mcg)	322	332	103	65	2039	317	328	101	65	2024	1025	7.5	5	0.0
Vitamin C (mg)	123	133	64	4	1006	125	135	64	4	1006	746	5.5	35	0.3
Vitamin D (mcg)	3.01	3.46	1.90	0.00	17.83	3.02	3.45	1.90	0.00	17.75	1119	8.2	90	0.7
Vitamin E (mg)	12.4	13.8	6.2	1.5	52.4	12.2	13.5	6.0	1.6	49.8	1863	13.7	123	0.9

For peer review only

Food group intake data from FETA

Average daily intakes for both men and women of the fourteen food groups readily available from FETA are shown in Table 3. Mean daily intakes of six of the food groups were higher in men than in women: alcohol, cereals, fats, meat, potatoes and sugars. However, women had higher intakes of fruit (278g v 212g) and vegetables (284g v 25~~1~~5g). Mean daily intakes of eggs, fish, milk, non-alcoholic beverages, nuts and seeds, and soups and sauces were similar in both men and women.

The effect of text matching in FETA

Tables 4 and 5 illustrate the variation in nutrient and food group intake data obtained in a random subset of 1 159 men and 1 340 women, respectively, depending on whether text matching of milks, breakfast cereals and baking and frying fats was applied. In general, mean nutrient intakes were higher when text matching was carried out. In men, (Table 4), quintile changes (>15%) were most evident in the following nutrients: Englyst fibre, polyunsaturated fat, folate, vitamin D and vitamin E. The food group "cereals and cereal products" was the only one of the fourteen groups where there was a difference, with 31 men moving 1 quintile.

In women, (Table 5), quintile changes (>15%) were also most evident in the same five nutrients. However, almost 21% of women also changed quintile for iron. Once again, the "cereals and cereal products" food group was the only food group where there was any difference, with 40 women moving 1 quintile.

For peer review only

Table 3 Average daily food group intakes for men (N=11 250) and women (N=13 602) participating in the EPIC-Norfolk study, from the FETA program

Food group	Men					Women				
	Median	Mean	SD	Minimum	Maximum	Median	Mean	SD	Minimum	Maximum
Alcoholic beverages (g)	101	204	315	0	2483	23	64	109	0	1728
Cereals & cereal products (g)	242	260	127	0	1456	215	231	110	0	1172
Eggs & egg dishes (g)	18	17	15	0	225	14	16	14	0	236
Fats & oils (g)	31	36	22	0	207	27	30	20	0	218
Fish & fish products (g)	32	37	26	0	362	32	38	26	0	309
Fruit (g)	179	212	164	0	2654	238	278	201	0	3742
Meat & meat products (g)	99	106	54	0	856	91	94	48	0	606
Milk & milk products (g)	407	420	182	0	1303	386	410	175	0	1560
Non-alcoholic beverages (g)	1157	1177	396	0	3707	1150	1165	403	0	4501
Nuts & seeds (g)	0	3	9	0	228	0	3	9	0	188
Potatoes (g)	125	122	69	0	1007	116	112	64	0	1506
Soups & sauces (g)	43	58	54	0	1004	43	57	53	0	1376
Sugars (g)	53	64	50	0	572	37	48	42	0	541
Vegetables (g)	236	255	123	0	2398	262	284	143	0	3539

For peer review only

Table 4 Comparison of average daily nutrient and food group intakes for men (N=1 159) participating in the EPIC-Norfolk study, from the FETA program, with and without the application of text matching

Formatted Table

	**FETA program, with text matching**					**FETA program, without text matching**								
Nutrient/Food group	Median	Mean	SD	Minimum	Maximum	Median	Mean	SD	Minimum	Maximum	Quintile change		Quintile change > 1	
											N	%	N	%
Energy (kcals)	2095	2176	678	658	7766	2091	2170	678	658	7787	28	2.4	0	0.0
Energy (kJs)	8822	9161	2848	2780	32555	8804	9138	2850	2780	32647	26	2.2	0	0.0
Protein (g)	82.8	85.0	22.8	22.1	272.3	82.5	84.7	22.8	22.1	272.3	34	2.9	0	0.0
Alcohol (g)	7.2	12.3	16.1	0.0	112.9	7.2	12.3	16.1	0.0	112.9	0	0.0	0	0.0
Carbohydrate (g)	261	270	93	63	1006	259	269	93	63	1003	48	4.1	0	0.0
Starch (g)	120	127	49	7	643	121	126	48	7	636	65	5.6	0	0.0
Englyst fibre (g)	17.5	18.3	6.6	3.6	71.8	17.3	17.9	6.3	3.6	64.5	198	17.1	10	0.9
Fat (g)	77.8	82.1	33.1	12.8	387.8	77.3	82.1	33.1	12.8	389.3	32	2.8	0	0.0
Monounsaturated fat (g)	26.5	28.2	12.2	3.5	131.1	26.7	28.7	12.5	3.7	138.7	88	7.6	0	0.0
Polyunsaturated fat (g)	13.5	14.9	7.3	3.0	67.0	12.7	14.1	6.8	3.0	60.7	179	15.4	17	1.5
Saturated fat (g)	30.1	31.8	14.1	3.3	160.0	30.3	32.2	14.3	3.3	160.3	72	6.2	1	0.1
Calcium (mg)	1015	1044	312	242	2848	1012	1044	313	242	2861	42	3.6	0	0.0
Iron (mg)	11.9	12.5	3.8	2.6	37.9	11.7	12.0	3.5	2.6	38.1	173	14.9	16	1.4
Potassium (mg)	3824	3889	957	1353	12675	3812	3873	951	1353	12551	52	4.5	0	0.0

For peer review only

Carotene (mcg)	3150	3348	1671	507	18295	3162	3353	1672	507	18338	6	0.5	0	0.0
Folate (mcg)	325	333	103	94	1222	316	326	101	94	1262	226	19.5	2	0.2
Vitamin C (mg)	105	113	55	17	619	104	112	55	17	619	22	1.9	0	0.0
Vitamin D (mcg)	3.08	3.64	2.17	0.03	16.40	3.06	3.64	2.19	0.03	20.52	227	19.6	8	0.7
Vitamin E (mg)	13.3	15.0	7.6	2.7	74.7	13.0	14.5	7.1	2.7	71.2	238	20.5	30	2.6
Alcoholic beverages (g)	104	201	301	0	1866	104	201	301	0	1866	0	0.0	0	0.0
Cereals & cereal products (g)	240	257	131	0	1378	238	255	130	0	1378	31	2.7	0	0.0
Eggs & egg dishes (g)	18	17	17	0	225	18	17	17	0	225	0	0.0	0	0.0
Fats & oils (g)	31	36	25	0	313	31	36	25	0	313	0	0.0	0	0.0
Fish & fish products (g)	32	37	25	0	153	32	37	25	0	153	0	0.0	0	0.0
Fruit (g)	184	216	158	0	1037	184	216	158	0	1037	0	0.0	0	0.0
Meat & meat products (g)	98	104	52	0	690	98	104	52	0	690	0	0.0	0	0.0
Milk & milk products (g)	414	428	187	0	1302	414	428	187	0	1302	0	0.0	0	0.0
Non-alcoholic beverages (g)	1159	1191	397	22	3677	1159	1191	397	22	3677	0	0.0	0	0.0
Nuts & seeds (g)	0	3	8	0	135	0	3	8	0	135	0	0.0	0	0.0
Potatoes (g)	125	121	78	0	1518	125	121	78	0	1518	0	0.0	0	0.0
Soups & sauces (g)	43	56	51	0	556	43	56	51	0	556	0	0.0	0	0.0
Sugars (g)	51	63	50	0	358	51	63	50	0	358	0	0.0	0	0.0
Vegetables (g)	238	256	128	15	1047	238	256	128	15	1047	0	0.0	0	0.0

For peer review only

Table 5 Comparison of average daily nutrient and food group intakes for women (N=1 340) participating in the EPIC-Norfolk study, from the FETA program, with and without the application of text matching

Formatted Table
Formatted: Right

Nutrient/Food group	FETA program, with text matching					FETA program, without text matching					Quintile change		Quintile change > 1	
	Median	Mean	SD	Minimum	Maximum	Median	Mean	SD	Minimum	Maximum	N	%	N	%
Energy (kcals)	1886	1946	607	608	8103	1880	1941	605	608	8134	50	3.7	0	0.0
Energy (kJs)	7938	8202	2554	2552	34410	7909	8177	2547	2552	34541	47	3.5	0	0.0
Protein (g)	80.3	82.5	22.2	26.8	277.0	79.9	82.1	22.1	26.8	276.6	43	3.2	0	0.0
Alcohol (g)	2.0	5.4	8.1	0.0	65.3	2.0	5.4	8.1	0.0	65.3	0	0.0	0	0.0
Carbohydrate (g)	238	250	90	67	1596	237	249	90	67	1603	58	4.3	0	0.0
Starch (g)	109	114	52	25	1288	108	114	52	25	1301	99	7.4	0	0.0
Englyst fibre (g)	18.6	19.3	7.4	4.1	103.7	17.8	18.7	7.1	3.3	97.2	247	18.4	13	1.0
Fat (g)	67.6	71.4	28.5	17.2	259.4	67.5	71.3	28.4	17.2	259.7	45	3.4	0	0.0
Monounsaturated fat (g)	22.7	24.4	10.6	4.8	104.2	23.1	24.6	10.6	4.8	103.8	133	9.9	0	0.0
Polyunsaturated fat (g)	12.2	13.6	6.2	2.6	42.5	11.5	12.9	5.9	2.5	39.4	224	16.7	11	0.8
Saturated fat (g)	25.2	27.2	12.4	5.1	109.6	25.5	27.5	12.4	5.1	109.6	74	5.5	2	0.1
Calcium (mg)	978	995	298	242	2528	976	992	297	242	2534	46	3.4	1	0.1
Iron (mg)	11.7	11.9	3.9	3.1	67.8	11.1	11.4	3.5	3.1	55.3	280	20.9	44	3.3
Potassium (mg)	3788	3874	994	1284	12702	3744	3848	987	1280	12526	68	5.1	0	0.0

For peer review only

Carotene (mcg)	3489	3731	1705	178	13796	3500	3736	1707	175	13796	11	0.8	0	0.0
Folate (mcg)	326	337	107	102	1311	318	329	105	97	1276	291	21.7	1	0.1
Vitamin C (mg)	124	133	63	4	809	122	132	62	4	809	34	2.5	0	0.0
Vitamin D (mcg)	3.07	3.49	1.89	0.22	12.06	3.02	3.46	1.89	0.29	12.46	248	18.5	9	0.7
Vitamin E (mg)	12.5	13.8	6.3	2.7	52.4	12.1	13.3	5.9	3.3	43.6	270	20.2	21	1.6
Alcoholic beverages (g)	21	61	104	0	1350	21	61	104	0	1350	0	0.0	0	0.0
Cereals & cereal products (g)	214	236	174	9	4948	212	234	174	9	4948	40	3.0	0	0.0
Eggs & egg dishes (g)	14	16	14	0	136	14	16	14	0	136	0	0.0	0	0.0
Fats & oils (g)	27	30	19	0	133	27	30	19	0	133	0	0.0	0	0.0
Fish & fish products (g)	32	39	26	0	187	32	39	26	0	187	0	0.0	0	0.0
Fruit (g)	238	277	199	0	2830	238	277	199	0	2830	0	0.0	0	0.0
Meat & meat products (g)	90	95	49	0	392	90	95	49	0	392	0	0.0	0	0.0
Milk & milk products (g)	381	410	174	0	959	381	410	174	0	959	0	0.0	0	0.0
Non-alcoholic beverages (g)	1148	1153	404	8	3215	1148	1153	404	8	3215	0	0.0	0	0.0
Nuts & seeds (g)	0	3	11	0	180	0	3	11	0	180	0	0.0	0	0.0
Potatoes (g)	116	113	61	0	785	116	113	61	0	785	0	0.0	0	0.0
Soups & sauces (g)	45	57	53	0	900	45	57	53	0	900	0	0.0	0	0.0
Sugars (g)	38	50	46	0	540	38	50	46	0	540	0	0.0	0	0.0
Vegetables (g)	265	288	140	2	1387	265	288	140	2	1387	0	0.0	0	0.0

For peer review only

DISCUSSION

FETA provides a new, freely available, standalone tool that can produce nutrient and food group intake values from data collected using the EPIC-Norfolk FFQ. It makes the EPIC-Norfolk FFQ readily accessible to end-users and enables them to process and analyse nutritional data. The data can either be entered into a spreadsheet, using the instructions provided, or by using the specifically developed Microsoft Access form-based entry tool. The Access entry tool allows easier entry without requiring knowledge of specific food codes. The software for FETA for Windows and Linux can be downloaded from the website, as can the Microsoft Access data entry utility (http://www.srl.cam.ac.uk/epic/epicffq/). Users are encouraged to register with EPIC-Norfolk, as this enables them to request assistance and support. The various types of output (with four levels of information) available should prove beneficial to researchers, especially those requiring more detailed information. There is an on-going need for information on the intake of food groups. While the data from either output 3 or 4 could be used to generate more detailed food group data, we have treated food groups as another type of nutrient – a pseudo-nutrient. The FETA input/look-up files can be easily modified to create new groups, greatly adding to the flexibility of the system for analysing food group consumption, while requiring no spreadsheet or programming skills on the part of the analyst. A helpful feature of FETA is the log file which documents errors relating to FFQ data and/or default food codes assigned.

FETA was designed and based on the extensively validated EPIC-Norfolk FFQ, originally developed in 1988, to assess the nutrient and food group intake of 40-79 year olds, who completed the FFQ between 1993 and 1997. The food list and look-up lists of milks, breakfast cereals and fats reflect this time period and the study population, as do the default milk, cereal, baking fat and frying fat codes assigned. However, the program was created in such a way that it can be customised for different study populations, easily enabled by the separation of the processing algorithm in the FETA program implementation from the data model text files. It is possible to delete/add foods and/or FFQ lines, and modify portion sizes as desired for a study.

For peer review only

Nutrient data may also be easily modified or added. It is also possible for FETA to be used with other questionnaires containing a different set of line items or different numbers of frequencies. Comparisons were carried out for a number of selected nutrients obtained from FETA and the previously validated CAFÉ program. These showed that the nutrient output from both programs were generally similar. –All differences (>0.1%) found from the comparison of detailed food/nutrient data at the individual level for 12 500 participants from FETA and the CAFÉ program can be explained by one or more of the following reasons: up to four cereal foods assigned by FETA, as compared to a maximum of two cereal foods assigned by CAFÉ; differences in default baking and frying fat codes assigned; correction for muesli portion size in cereal data; exclusion of porridge from cereal data (free text); default codes assigned for milk, cereals or fats to participants using FETA (where no food codes were assigned by CAFÉ program); rounding error (only where percentage absolute differences were between 0.1 to 1%) and changes made to the nutrient data of six of the nine new foods as well as to the default code for milk. A section entitled 'What are the differences between FETA versus CAFÉ processing?' found at http://www.srl.cam.ac.uk/epic/epicffq/FAQs.html further explains the aforementioned differences.

Although nutrient intakes as calculated by FETA and CAFÉ were similar, some relatively small differences existed, but these and the quintile shift of men and women can be explained. In FETA, a number of changes were made to the processing of breakfast cereals, affecting carbohydrate, starch, Englyst fibre, iron and folate estimates. The vitamin C content per 100g of low calorie/diet fizzy drinks was changed from 5 to 0 mg and the vitamin E content of crunchy oat cereal and oil and fat non-specific was increased. Changes made to the processing of fats in Questions 6 and 7 in Part 2 of the FFQ, in addition to changes made to the fatty acid profile of the three new fats, could help explain the small differences observed in monounsaturated, polyunsaturated and saturated fat intakes.

For peer review only

There was quite a large range in intake in the fourteen food groups, with a minimum intake of zero for each of the food groups. It is difficult to compare food group intake data as the groupings of foods often varies. However, the combined mean intake of fruit (excluding juices) and vegetables for men and women was 467g and 562g respectively, achieving the Government's 'Five a day' recommendation(23), using a portion size of 80 g.

Whilst text matching only affected one food group (cereals and cereal products), more than 15% of men and women changed quintile for a number of nutrients: Englyst fibre, polyunsaturated fat, folate, vitamin D and vitamin E, and iron (women only). Yet again, these nutrients related to the text matching of breakfast cereals and baking and frying fats. The inclusion of these data illustrates the effect of text matching on the ranking of individuals for certain nutrients and will enable future researchers using FETA to make informed decisions on the benefit of text matching for their study.

We have not addressed or discussed common FFQ issues, such as the number of items in a food list or the use of a single average portion size, as these are not the focus of this paper and have been reviewed previously (24,25).

It is anticipated that future updates of FETA might contain a number of improvements and overcome some of the limitations of FETA, currently released as version 2.53 for Windows and Linux (last updated 15/03/2013 and 21/02/2013 respectively). The source code has been made available online which enables users to make modifications and improvements to the program. Currently, we have made available Windows and Linux versions and it is hoped that an OS X version will follow soon. We are currently working on a Libreoffice version of the Microsoft Access form-based entry tool.

In conclusion, we have created a new, open source, standalone, cross-platform FFQ processing tool, FETA, to produce nutrient and food group data for researchers using the EPIC-Norfolk FFQ. The tool produces similar nutrient and food group values to the previously validated CAFÉ program, but is more accessible. Although FETA was designed and based on the EPIC-Norfolk

For peer review only - http://bmjopen.bmj.com/site/about/guidelines.xhtml

FFQ, the program was created in such a way that it can be customised for different study populations. It is anticipated that the development and availability of FETA will be a useful addition to the field of nutritional epidemiology and dietary public health.

Acknowledgments

We thank Mr Adam Dickinson, senior data manager at the MRC Epidemiology Unit, and his team members for their contribution to project management of FETA; Professor Nick Wareham, as EPIC-Norfolk study PI; and Mr Jamal Natour, as FETA software developer. The authors would also like to thank all the participants of the EPIC-Norfolk study and the EPIC-Norfolk staff for their help with this work.

Contributors

AAM contributed to the software development, assisted in statistical analyses and drafted the manuscript. AB and RL contributed to the software development, assisted in statistical analyses and contributed to the manuscript. DJP-S wrote the step-based graphical wizard for running FETA and contributed to the manuscript. NGF, LO'C and K-TK (Principal Investigator of EPIC-Norfolk) contributed to the manuscript. APK created the Microsoft Access form-based entry tool and contributed to the manuscript. All authors approved the final manuscript.

Funding

This study was supported by programme grants from the MRC Population Health Sciences Research Network (PHSRN), Cancer Research UK (C864/A8257) and the Medical Research Council (G0401527 and G1000143); NGF was supported by the Medical Research Council (MC_UP_A100_1003); APK is funded by a Wellcome Trust Clinical Research Fellowship.

Competing interests None.

Ethics approval Norwich Local Research Ethics Committee.

References

For peer review only

1. Subar AF. Developing dietary assessment tools. J Am Diet Assoc 2004 May;**104**(5):769–70. www.ncbi.nlm.nih.gov/pubmed/15127062 (accessed 5 Nov 2013).

2. Welch AA, Luben R, Khaw KT, et al. The CAFE computer program for nutritional analysis of the EPIC-Norfolk food frequency questionnaire and identification of extreme nutrient values. J Hum Nutr Diet 2005;**18**(2):99–116.

3. Bingham SA, Gill C, Welch A, et al. Validation of dietary assessment methods in the UK arm of EPIC using weighed records, and 24-hour urinary nitrogen and potassium and serum vitamin C and carotenoids as biomarkers. Int J Epidemiol 1997;**26**(1):S137–S151.

4. Bingham SA, Welch AA, McTaggart A, et al. Nutritional methods in the European Prospective Investigation of Cancer in Norfolk. Public Health Nutr 2001;**4**(3):847–58.

5. McKeown NM, Day NE, Welch AA, et al. Use of biological markers to validate self-reported dietary intake in a random sample of the European Prospective Investigation into Cancer United Kingdom Norfolk cohort. Am J Clin Nutr 2001;**74**:188–96.

6. Rimm EB, Giovannucci EL, Stampfer MJ, et al. Reproducibility and validity of an expanded self-administered semiquantitative food frequency questionnaire among male health professionals. Am J Epidemiol 1992 May;**135**(10):1114–26. www.ncbi.nlm.nih.gov/pubmed/1632423 (accessed 5 Nov 2013).

7. Bingham SA, Gill C, Welch A, et al. Comparison of dietary assessment methods in nutritional epidemiology: weighed records v. 24 h recalls, food-frequency questionnaires and estimated-diet records. Br J Nutr 1994;**72**(4):619–43.

8. Day N, Oakes S, Luben R, et al. EPIC-Norfolk: study design and characteristics of the cohort. European Prospective Investigation of Cancer. Br J Cancer 1999 Jul;**80**(Suppl 1):95–103. www.ncbi.nlm.nih.gov/pubmed/10466767 (accessed 5 Nov 2013).

For peer review only

9. Gregory J, Foster K, Tyler H, Wiseman M. The Dietary and Nutritional Survey of British Adults. London: Her Majesty's Stationary Office (HMSO) 1990.

10. MAFF. The Dietary and Nutritional Survey of British Adults - Further Analysis. London: Her Majesty's Stationary Office (HMSO) 1994.

11. MAFF. Food Portion Sizes. 2nd ed. London: Her Majesty's Stationary Office (HMSO) 1993.

12. Holland B, Unwin I, Buss D. Cereals and cereal products. The third supplement to McCance & Widdowson's The Composition of Foods (4th Edition). Cambridge: RSC/MAFF 1988.

13. Holland B, Unwin I, Buss D. Milk products and eggs. The fourth supplement to McCance & Widdowson's The Composition of Foods (4th Edition). Cambridge: RSC/MAFF 1989.

14. Holland B, Welch AA, Unwin D, Buss DH, Paul AA, Southgate DAT. McCance and Widdowson's The Composition of Foods. Cambridge: Royal Society of Chemistry (RSC) 1991.

15. Holland B, Unwin I, Buss D. Vegetables, herbs and spices. The fifth supplement to McCance & Widdowson's The Composition of Foods (4th Edition). Cambridge: RSC/MAFF 1991.

16. Holland B, Unwin I, Buss D. Fruit and nuts. The first supplement to McCance & Widdowson's The Composition of Foods (5th Edition). Cambridge: RSC/MAFF 1992.

17. Holland B, Welch A, Buss D. Vegetable dishes. The second supplement to McCance & Widdowson's The Composition of Foods (5th Edition). Cambridge: RSC/MAFF 1992.

For peer review only

18. Holland B, Brown J, Buss D. Fish and fish products. The third supplement to McCance & Widdowson's The Composition of Foods (5th Edition). Cambridge: RSC/MAFF 1993.

19. Chan W, Brown J, Buss D. Miscellaneous foods. The fourth supplement to McCance & Widdowson's The Composition of Foods (5th Edition). Cambridge: RSC/MAFF 1994.

20. Chan W, Brown J, Lee S, Buss D. Meat, poultry and game. The fifth supplement to McCance & Widdowson's The Composition of Foods (5th Edition). Cambridge: RSC/MAFF 1995.

21. Chan W, Brown J, Church S, Buss D. Meat products and dishes. The sixth supplement to McCance & Widdowson's The Composition of Foods (5th Edition). Cambridge: RSC/MAFF 1996.

22. COMA. Dietary Reference Values for Food Energy and Nutrients for the United Kingdom. London: Her Majesty's Stationary Office (HMSO) 1991.

23. Department of Health. www.nhs.uk/Livewell/5ADAY/Pages/5ADAYhome.aspx (accessed 5 Nov 2013).

24. Cade JE, Burley VJ, Warm DL, et al. Food-frequency questionnaires: a review of their design, validation and utilisation. Nutr Res Rev 2004 Jun;**17**(1):5–22. www.ncbi.nlm.nih.gov/pubmed/19079912 (accessed 30 Oct 2013).

25. Molag ML, de Vries JHM, Ocké MC, et al. Design characteristics of food frequency questionnaires in relation to their validity. Am J Epidemiol 2007 Dec;**166**(12):1468–78. www.ncbi.nlm.nih.gov/pubmed/17881382 (accessed 5 Nov 2013).

For peer review only

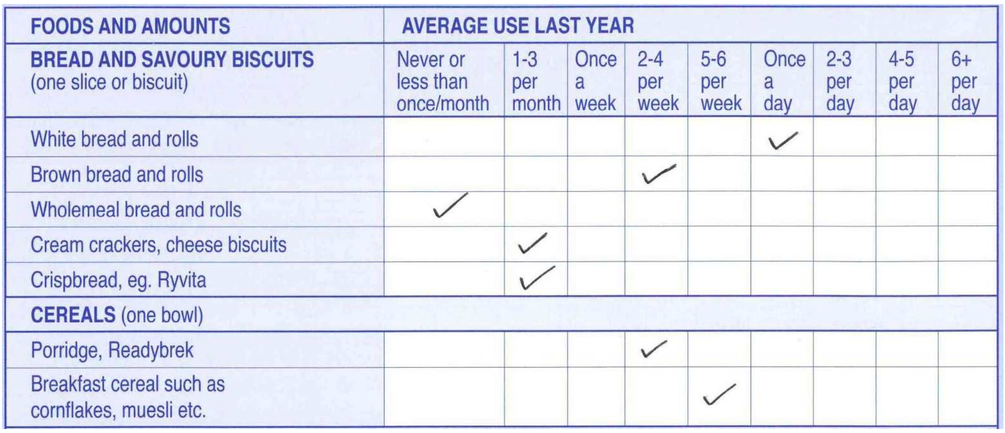

FOODS AND AMOUNTS	AVERAGE USE LAST YEAR								
**BREAD AND SAVOURY BISCUITS** (one slice or biscuit)	Never or less than once/month	1-3 per month	Once a week	2-4 per week	5-6 per week	Once a day	2-3 per day	4-5 per day	6+ per day
White bread and rolls						✓			
Brown bread and rolls				✓					
Wholemeal bread and rolls	✓								
Cream crackers, cheese biscuits		✓							
Crispbread, eg. Ryvita		✓							
**CEREALS** (one bowl)									
Porridge, Readybrek				✓					
Breakfast cereal such as cornflakes, muesli etc.					✓				

Part 1 (main part) of the EPIC-Norfolk FFQ, illustrating bread, savoury biscuits and breakfast cereals
191x81mm (300 x 300 DPI)

For peer review only

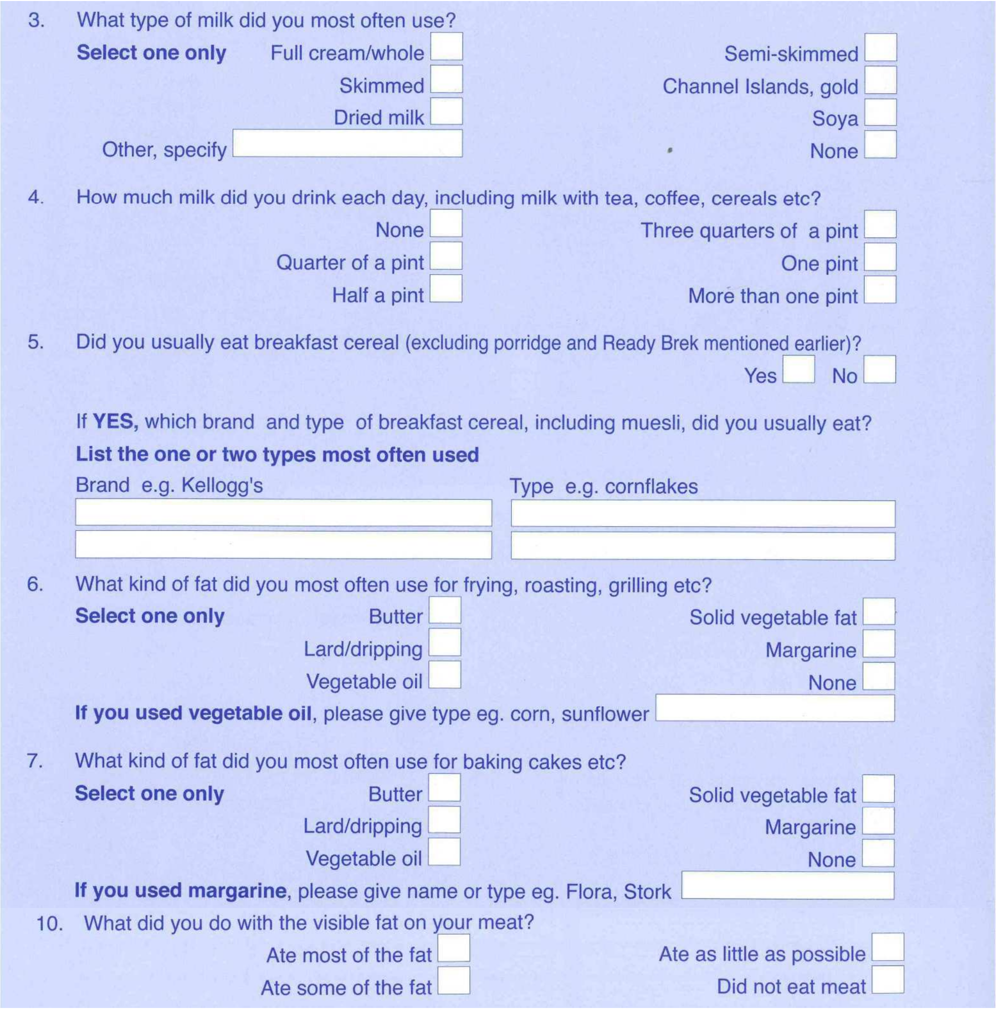

3. What type of milk did you most often use?

Select one only

- Full cream/whole ☐
- Semi-skimmed ☐
- Skimmed ☐
- Channel Islands, gold ☐
- Dried milk ☐
- Soya ☐
- Other, specify ______
- None ☐

4. How much milk did you drink each day, including milk with tea, coffee, cereals etc?

- None ☐
- Three quarters of a pint ☐
- Quarter of a pint ☐
- One pint ☐
- Half a pint ☐
- More than one pint ☐

5. Did you usually eat breakfast cereal (excluding porridge and Ready Brek mentioned earlier)?

Yes ☐ No ☐

If **YES,** which brand and type of breakfast cereal, including muesli, did you usually eat?

List the one or two types most often used

Brand e.g. Kellogg's	Type e.g. cornflakes

6. What kind of fat did you most often use for frying, roasting, grilling etc?

Select one only

- Butter ☐
- Solid vegetable fat ☐
- Lard/dripping ☐
- Margarine ☐
- Vegetable oil ☐
- None ☐

If you used vegetable oil, please give type eg. corn, sunflower ______

7. What kind of fat did you most often use for baking cakes etc?

Select one only

- Butter ☐
- Solid vegetable fat ☐
- Lard/dripping ☐
- Margarine ☐
- Vegetable oil ☐
- None ☐

If you used margarine, please give name or type eg. Flora, Stork ______

10. What did you do with the visible fat on your meat?

- Ate most of the fat ☐
- Ate as little as possible ☐
- Ate some of the fat ☐
- Did not eat meat ☐

Questions from part 2 of the EPIC-Norfolk FFQ, used by FETA
207x208mm (300 x 300 DPI)

Appendix 1 Extract from a sample log file produced during the processing of 10 ids, using output 1.

2013-01-29 11:54 am: Note : Starting database setup

2013-01-29 11:54 am: Note : Loading imports for 'foods' completed

2013-01-29 11:54 am: Note : Loading imports for 'meals' completed

2013-01-29 11:54 am: Note : Loading imports for 'nutrients' completed

2013-01-29 11:54 am: Note : Loading imports for 'food_nutrients' completed

2013-01-29 11:54 am: Note : Loading imports for 'meal_foods' completed

2013-01-29 11:54 am: Note : Loading imports for 'weights' completed

2013-01-29 11:54 am: Note : Loading imports for 'portions' completed

2013-01-29 11:54 am: Note : Loading imports for 'frequencies' completed

2013-01-29 11:54 am: Note : Loading imports for 'cereals' completed

2013-01-29 11:54 am: Note : Loading imports for 'milks' completed

2013-01-29 11:54 am: Note : Completed database setup

2013-01-29 11:54 am: Error: Respondent: 001A supplied invalid frequency: -9 for meal: BURGER

2013-01-29 11:54 am: Error: Respondent: 001A supplied invalid frequency: -9 for meal: LIVER

2013-01-29 11:54 am: Error: Respondent: 003C supplied no baking fat food_codes

For peer review only

For peer review only - http://bmjopen.bmj.com/site/about/guidelines.xhtml

18 2013-01-29 11:54 am: Note : Respondent: 003C using default baking fat code: 17018

19 2013-01-29 11:54 am: Error: Respondent: 004D supplied invalid frequency: -9 for meal: FRUIT_SQUASH

20 2013-01-29 11:54 am: Error: Respondent: 005E supplied invalid frequency: -9 for meal: CHICKEN

21 2013-01-29 11:54 am: Error: Respondent: 005E supplied no frying fat food_codes

22 2013-01-29 11:54 am: Note : Respondent: 005E using default frying fat code: 17046

23 2013-01-29 11:54 am: Error: Respondent: 008H supplied invalid frequency: -9 for meal: INSTANT_COFFEE

24 2013-01-29 11:54 am: Error: Respondent: 008H supplied no baking fat food_codes

25 2013-01-29 11:54 am: Note : Respondent: 008H using default baking fat code: 17018

26 2013-01-29 11:54 am: Error: Respondent: 009J supplied invalid frequency: -9 for meal: DAIRY_DESSERT

27 2013-01-29 11:54 am: Error: Respondent: 009J supplied invalid frequency: -4 for meal: EGGS

28 2013-01-29 11:54 am: Error: Respondent: 009J supplied invalid frequency: -9 for meal: LOWCAL_SALAD_CREAM

29 2013-01-29 11:54 am: Error: Respondent: 009J supplied invalid frequency: -9 for meal: PLAIN_BISCUIT

30 2013-01-29 11:54 am: Error: Respondent: 009J supplied invalid frequency: -9 for meal: INSTANT_COFFEE

31 2013-01-29 11:54 am: Error: Respondent: 009J supplied invalid frequency: -9 for meal: COFFEE_WHITENER

32 2013-01-29 11:54 am: Error: Respondent: 009J supplied invalid frequency: -9 for meal: SPINACH

33 2013-01-29 11:54 am: Error: Respondent: 009J supplied no visible fat weighting

34 2013-01-29 11:54 am: Note : Respondent: 009J using default weighting: 1

For peer review only

2013-01-29 11:54 am: Error: Respondent: 010K supplied no visible fat weighting

2013-01-29 11:54 am: Note : Respondent: 010K using default weighting: 1

2013-01-29 11:54 am: Note : Processing completed for Respondent: 001A

2013-01-29 11:54 am: Note : Processing completed for Respondent: 002B

2013-01-29 11:54 am: Note : Processing completed for Respondent: 003C

2013-01-29 11:54 am: Note : Processing completed for Respondent: 004D

2013-01-29 11:54 am: Note : Processing completed for Respondent: 005E

2013-01-29 11:54 am: Note : Processing completed for Respondent: 006F

2013-01-29 11:54 am: Note : Processing completed for Respondent: 007G

2013-01-29 11:54 am: Note : Processing completed for Respondent: 008H

2013-01-29 11:54 am: Note : Processing completed for Respondent: 009J

2013-01-29 11:54 am: Note : Processing completed for Respondent: 010K

2013-01-29 11:54 am: Note : Questionaire: sample_input_290113.csv processing completed successfully, processed(10) respondents

For peer review only

For peer review only - http://bmjopen.bmj.com/site/about/guidelines.xhtml
